# Bicistronic Lentiviral Architecture Increases Surface Density of Membrane-Associated HIV Entry Inhibitors and Confers Tropism-Independent Protection to Modified Cells

**DOI:** 10.3390/cells15181704

**Published:** 2026-09-19

**Authors:** Yaroslava V. Chervyakova, Alena V. Smirnova, Andrei E. Siniavin, Vasilisa E. Soldatova, Diana D. Gamzik, Anna V. Tvorogova, Islam M. Nakastoev, Alexander V. Filatov, Natalia A. Kruglova, Alexandra Konstantia Y. Maslennikova

**Affiliations:** 1 Institute of Gene Biology, Russian Academy of Sciences, Ministry of Science and Higher Education of the Russian Federation, Moscow 119334, Russia; chervyakovayaroslava@gmail.com (Y.V.C.); smirnova.av97@mail.ru (A.V.S.); april.vasilisa@gmail.com (V.E.S.); annatvor@mail.ru (A.V.T.); natalya.a.kruglova@yandex.ru (N.A.K.); 2 Federal State Budget Institution “National Research Centre for Epidemiology and Microbiology Named After Honorary Academician N. F. Gamaleya”, The Ministry of Health of the Russian Federation, Moscow 123089, Russia; andreysi93@ya.ru; 3 Zoological Institute of the Russian Academy of Sciences, Saint Petersburg 199034, Russia; diana.gamzik@mail.ru; 4 Federal State Budgetary Institution National Medical Research Centre for Hematology, The Ministry of Health of the Russian Federation, Moscow 125167, Russia; slep06@gmail.com; 5 National Research Center Institute of Immunology, Federal Medical Biological Agency of Russia, Moscow 115522, Russia; avfilat@yandex.ru

**Keywords:** gene therapy, HIV fusion inhibitor, CD4^+^ T lymphocytes, hematopoietic stem cells, ex vivo cell engineering, lentiviral delivery, GPI-anchored C-peptides, HIV resistance, inducible expression system

## Abstract

**Background:** Previous studies have demonstrated the efficacy of membrane-anchored C-peptides against HIV and have shown that the surface expression level of these peptides is critical for effective protective activity. **Methods:** Bicistronic genetic constructs were designed for GPI-anchored expression of protective C-peptides on the cell membrane. The constructs were delivered using lentiviral vectors and tested in primary CD4^+^ lymphocytes and hematopoietic stem cells. **Results:** The bicistronic configuration significantly enhanced surface expression levels compared to monocistronic constructs and allowed simultaneous expression of two distinct protective peptides, thereby potentiating their activity. Lentiviral vectors harboring these constructs conferred robust protection to primary CD4^+^ lymphocytes against HIV infection irrespective of viral tropism and provided a selective advantage to transduced cells upon challenge with replication-competent HIV strains. The bicistronic construct was also suitable for lentiviral transduction of hematopoietic stem cells. **Conclusions:** These findings establish bicistronic GPI-anchored C-peptide expression as a promising platform for generating HIV-resistant immune cells and highlight its potential for ex vivo gene therapy.

## 1. Introduction

Human immunodeficiency virus type 1 (HIV) remains a significant public health challenge, as current antiretroviral therapies require lifelong administration and cannot eradicate latent reservoirs. A critical barrier to durable cure is the emergence of drug-resistant viral strains, which compromises treatment efficacy and accelerates disease progression. Gene therapy offers a promising alternative by engineering immune cells to resist HIV infection [1]. The feasibility of this approach was established by the Berlin [2,3,4] and London [5,6] patients, who achieved sustained virologic remission following allogeneic hematopoietic stem cell (HSC) transplantation from donors carrying the homozygous *CCR5Δ32* mutation. These cases demonstrated that targeted modification of host susceptibility can lead to functional cure.

Subsequent efforts have explored CRISPR/Cas9 [1] and TALEN-based [7] knockout of HIV coreceptor genes (*CCR5* and *CXCR4*). However, several limitations constrain clinical translation. Knockout of *CCR5* alone does not protect against CXCR4-tropic viruses, permitting potential viral rebound [1,8]. Simultaneous biallelic disruption of both coreceptors remains technically challenging and insufficiently efficient for widespread use [9,10]. Furthermore, multiplexed guide RNA approaches increase off-target mutagenesis risks, and *CXCR4* ablation is restricted to post-thymic lymphocytes because CXCR4 signaling is essential for hematopoietic progenitor maturation [11,12]. Strategies targeting proviral DNA excision hold theoretical appeal but fail unless modified cells are also protected against reinfection.

Among classical gene-addition gene therapy strategies, membrane-displayed fusion inhibitors and restriction factors such as TRIM5α [13,14,15] have attracted considerable attention. Genetically encoded peptides derived from the heptad repeat 2 (HR2) domain of gp41, termed C-peptides, provide the broadest spectrum of entry inhibition among all tested gene therapy strategies [16,17,18,19]. The HIV protein gp41 is the transmembrane subunit of the HIV envelope glycoprotein complex (Env). Together with the surface subunit gp120, it mediates virus–host cell attachment and subsequent fusion of the viral envelope with the host cell plasma membrane. Binding to the receptor (CD4) and coreceptor (CCR5 or CXCR4) is mediated primarily by gp120, whereas gp41 drives the membrane fusion step itself. C-peptides bind the N-terminal heptad repeat of gp41, blocking six-helix bundle formation required for viral–cell membrane fusion. In our previous study, a panel of HR2-derived C-peptides was screened for anti-HIV activity when displayed within the minimal human GPI-anchored protein CD52; among them, MT-C34 and 2P23 emerged as the most potent inhibitors [19]. Structurally, T20 comprises the full-length 36-amino-acid HR2 sequence of gp41, whereas MT-C34 is a C34 variant bearing an N-terminal M-T hook (Met-Thr) that caps the deep hydrophobic pocket on the gp41 NHR trimer and stabilizes inhibitor binding [20,21]. In contrast, 2P23 is a synthetic 23-residue peptide designed on the basis of the M-T hook structure, containing the hook residues and primarily targeting the gp41 NHR pocket site; its shortened length confers enhanced proteolytic stability and improved membrane accessibility when displayed within GPI-anchored scaffolds [19,21,22]. The amino-acid sequences of these three peptides are aligned in Supplementary Figure S1.

Enfuvirtide (T20) is the first HR2-derived peptide approved by the FDA for clinical use against HIV infection. Although it demonstrates clinical utility, its soluble formulation suffers from poor pharmacokinetics, injection-site reactions, and rapid selection of escape mutants [20]. Current lentiviral delivery platforms display C-peptides either fused to CXCR4 protein [21] or anchored via large GPI-linked proteins such as LNGFR or DAF [22], which localize to lipid rafts near HIV coreceptors [19]. Since C-peptides require access to gp41 following coreceptor engagement and membrane apposition, their polypeptide length and spatial positioning relative to the plasma membrane may critically dictate inhibitory potency.

Previous screening identified MT-C34 and 2P23 as the most potent C-peptides when displayed within the minimal human GPI-anchored protein CD52 [19]. Both constructs conferred equivalent protection in HEK293T/CD4/R5 cells; however, 2P23 exhibited reduced activity in the T-lymphoblastic cell line CCRF-CEM/CCR5 (CEM/R5) due to lower surface expression. Comparative analysis revealed that CRISPR/Cas9-mediated knock-in achieved higher peptide density than lentiviral transduction, yielding superior protection of primary CD4^+^ T cells. Nevertheless, knock-in efficiency remained suboptimal for clinical application, while electroporation induced substantial CD4^+^ T-cell mortality [19]. Consequently, we prioritized lentiviral transduction for further development.

In this study, we engineered optimized bicistronic lentiviral vectors expressing two identical or distinct C-peptides within the CD52 context to enhance surface density and reduce resistance emergence. We validated these constructs using primary CD4^+^ T lymphocytes challenged with replication-competent HIV strains with different tropisms, including an enfuvirtide-resistant variant harboring a V38A envelope mutation. The vectors efficiently transduced HSCs without compromising stemness markers (CD34, CD90, CD133, CD38). Additionally, we integrated a CXCR4-mediated homing enhancement element with an inducible regulatory system to enable conditioning-independent engraftment of genetically modified CD34^+^ HSCs. This inducible control is expected to prevent sustained receptor hyperactivation, thereby mitigating the risk of Warts, Hypogammaglobulinemia, Infections, and Myelokathexis (WHIM) syndrome-associated complications. Here, we report the design, optimization, and functional validation of these lentiviral vectors, demonstrating their capacity to confer tropism-independent protection, preserve cellular physiology, and support niche-enrichment-based HSC homing strategies.

## 2. Materials and Methods

### 2.1. Cell Lines and Primary Cells

HEK293T cells were obtained from the NIH AIDS Research and Reference Reagent Program. The CCRF-CEM (CD4^+^ T-cell) and Raji (B-cell) lines were acquired from ATCC. HEK293T and CCRF-CEM cells stably transfected with CD4 and CCR5 were obtained by us previously (HEK293T/CD4/R5 and CEM/R5, respectively) [19]. Peripheral blood mononuclear cells (PBMCs) were isolated from fresh whole blood or leukocyte-rich buffy coats using Ficoll-Paque density gradient centrifugation (Paneco, Moscow, Russia). CD4^+^ T lymphocytes were purified using a magnetic separation kit (130-096-533; Miltenyi Biotec, Germany). Isolated CD4^+^ T cells were activated with anti-CD3/anti-CD28 magnetic beads (L00899-0.5; GenScript, Piscataway, NJ, USA) according to the manufacturer’s protocol and cultured in medium supplemented with 100 IU/mL recombinant human IL-2 (NPK Biotech, Saint Petersburg, Russia). CD34^+^ HSCs were isolated by magnetic enrichment (L00967-1; GenScript, Piscataway, NJ, USA) from cryopreserved mobilized peripheral blood samples obtained from donors under a cooperation agreement with the National Medical Research Center for Hematology (Moscow, Russia). Biomaterials unclaimed for clinical use were utilized following institutional approval.

HEK293T cells were maintained in high-glucose DMEM (Paneco, Moscow, Russia) supplemented with 10% fetal bovine serum (FBS), 2 mM glutamine, and 40 μg/mL gentamicin. CCRF-CEM, Raji, PBMCs, and primary CD4^+^ T cells were cultured in RPMI 1640 (R0883; Sigma-Aldrich, St. Louis, MI, USA) containing 10% FBS, 2 mM glutamine, and 40 μg/mL gentamicin. CD34^+^ HSCs were expanded in StemSpan (#09605; Stem Cell Technologies, Vancouver, BC, Canada) supplemented with stem cell factor (SCF) (100 ng/mL), Flt3 ligand (FL) (100 ng/mL), recombinant human thrombopoietin (TPO) (100 ng/mL), and interleukin-6 (IL-6) (60 ng/mL) (all cytokines: Miltenyi Biotec, Bergisch Gladbach, Germany). All procedures involving human biomaterials complied with and were approved by the local Institutional Review Board. Written informed consent was obtained from all donors.

### 2.2. Plasmid Construction

Third-generation lentiviral vectors for the stable expression of HIV fusion-inhibiting peptides were created based on the plasmid pCDH511b CMV hACE2/EF1α eGFP [23], kindly provided by Prof. Dr. A.G. Gabibov. To enhance transgene expression, our lentiviral transfer vectors incorporate the Woodchuck hepatitis virus post-transcriptional regulatory element (WPRE), a cis-acting RNA element that increases mRNA stability and translational efficiency across diverse cell types. In accordance with standard practice for clinically relevant vectors, we used the oncogenicity-reduced WPRE variant, in which the sequence elements associated with residual transforming potential have been mutated while the RNA-stabilizing and translational-enhancing functions of WPRE are preserved. The full-length hACE2 gene under the control of the CMV promoter was replaced by the mClover-smAID-P2A-MT-C34_CD52_ or mClover-smAID-P2A-2P23_CD52_ construct from the plasmid pUCHR-mClover-smAID-P2A-2P23_CD52_ [19] under the control of the EF1α promoter, replacing the GFP sequence. Subsequently, a portion of the mClover-smAID was replaced with either MT-C34_CD52_ or 2P23_CD52_. To obtain monocistronic vectors, the MT-C34_CD52_ or 2P23_CD52_ sequences were cloned into the plasmid in place of the mClover-smAID-P2A-2P23_CD52_ construct. Thus, a panel of lentiviral vectors was constructed: pCDH-2P23_CD52_-MT-C34_CD52_, pCDH-MT-C34_CD52_-MT-C34_CD52_, pCDH-2P23_CD52_-2P23_CD52_, pCDH-MT-C34_CD52_, and pCDH-2P23_CD52_. A detailed description of the gene therapy sequences obtained is presented in Supplementary Table S1.

The accuracy of all sequences was confirmed by sequencing. To generate the plasmids pCDH-CMV-2P23-CXCR4-GFP and pCDH-CMV-CXCR4, the corresponding sequences were cloned under the control of the CMV promoter of the pCDH511b CMV hACE2/EF1α eGFP plasmid, replacing the full-length hACE2 gene. To obtain the plasmid pCDH-2P23-CXCR4-MT-C34_CD52_, the MT-C34_CD52_ sequence in pCDH-MT-C34_CD52_-MT-C34_CD52_ was replaced with the 2P23-CXCR4 sequence. The HIV molecular clones pNL4-3 and pNL(AD8) were kindly provided by Eric Freed (NCI-Frederick, USA). The mutant NL4-3 in the gp41 region was described previously [19]. The vector expressing the gRNA, pKS gRNA BB, was described previously [24]. The plasmid for wild-type spCas9 expression, pcDNA3.3-hCas9 (Addgene #41815), was also used.

### 2.3. Lentivirus Production

Lentiviral pseudotypes were generated by transient transfection of HEK293T cells using Lipofectamine 2000. Cells were transfected with a mixture of the packaging plasmids pMDLg/pRRE (Addgene #12251) and pRSV-Rev (Addgene #12253), kindly provided by Prof. Dr. A.G. Gabibov, together with pCMV-VSV-G (Addgene #8454) and the respective transfer vector in a mass ratio of 2:1:1:1.3. Viral supernatants were harvested 54 h post-transfection, clarified by filtration (0.45 μm), and concentrated by ultracentrifugation. Viral titers were quantified by measuring HIV capsid antigen (p24) levels via ELISA (D-0134; Vector Best, Novosibirsk, Russia). Replication competence was assessed by culturing transduced HEK293T cells for 28 days; no viral replication was detected, as evidenced by undetectable levels of p24 in the culture supernatants.

### 2.4. Generation of Model Cell Lines

CEM/R5 cells [19] were transduced with the respective lentiviral vectors at a multiplicity of infection (MOI) less than 0.3. Transduced cells were stained with monoclonal antibodies specific to the target peptide(s) [19] and purified by fluorescence-activated cell sorting (FACS) to achieve ≥98% population homogeneity.

For generation of the *CXCR4* knockout CEM/R5 cell line, 2 × 10^6^ cells were electroporated using the Neon system (Invitrogen/Thermo Fisher Scientific, Waltham, MA, USA). The electroporation mixture consisted of cells, 1 μg of the pKS gRNA BB plasmid expressing a guide RNA targeting exon 2 of the *CXCR4* gene (protospacer: 5′-CACTTCAGATAACTACACCG-3′, PAM: AGG) [19], and 3 μg of the pcDNA3.3-hCas9 expression plasmid. Following electroporation, cells were cultured under standard conditions and subsequently screened for loss of surface CXCR4 expression by flow cytometry.

### 2.5. Quantitative Real-Time PCR (qPCR)

To compare the transgene integration efficiency between the bicistronic and monocistronic constructs, genomic DNA was isolated, and transgene copy numbers were quantified by qPCR using the Delta Delta (ΔΔCt) method, normalized to the reference gene *BMP4*. The monocistronic construct was used as the calibrator, and the bicistronic construct was analyzed as the test sample. Pairwise comparisons were performed to assess relative copy numbers between the two constructs. All primer sequences are detailed in Supplementary Table S2.

### 2.6. Lentiviral Transduction of Primary Cells

Twenty-four h following activation with anti-CD3/anti-CD28 magnetic nanobeads, CD4^+^ T lymphocytes were seeded at a density of 2.5 × 10^6^ cells/mL in complete culture medium. Lentiviral supernatant was added at an MOI of 10. After a 6-h incubation, the culture volume was adjusted to achieve a final cell density of 1 × 10^6^ cells/mL. The medium was replenished after 24 h, and cells were expanded to a density of 1 × 10^6^ cells/mL.

Following a 24-h pre-incubation with growth factors, CD34^+^ HSCs were seeded at a density of 2.5 × 10^5^ cells/mL. Cells were transduced with lentivirus at varying MOIs (10, 20, or 40). Following a 24-h incubation, the medium was replaced with fresh cytokine-supplemented medium, and cells were expanded to a density of 1 × 10^6^ cells/mL.

### 2.7. Flow Cytometry, Cell Sorting, and Cytokine Quantification

For surface staining, live cells were washed once with PBS and incubated with primary antibodies specific to C-peptides (anti-C24 and/or anti-2P23) [19] in PBS for 20 min at room temperature. Cells were then washed twice with PBS, incubated with fluorophore-conjugated secondary antibodies (5 μg/mL) for 20 min, and subjected to a final PBS wash.

For intracellular staining, cells were fixed in 4% paraformaldehyde (Sigma-Aldrich, St. Louis, MO, USA) in PBS for 15 min, washed, and permeabilized for 30 min in PBS containing 0.1% saponin (Sigma-Aldrich, St. Louis, MO, USA) and 2% normal mouse serum. HIV Gag p24 was detected using rhodamine-conjugated monoclonal antibody KC57-RD1 (6604667; Beckman Coulter, Indianapolis, IN, USA) diluted 1:200 for 40 min. Following three washes with permeabilization buffer, cells were resuspended in PBS prior to acquisition.

The following mouse monoclonal antibodies were employed: CD4-Alexa Fluor 488 (E-AB-F1109L), CD25-PE (E-AB-F1102D), CD34-APC (E-AB-F1143E), CD90-FITC (E-AB-F1167C), CD133-PE (E-AB-F1268D), CCR5-Alexa Fluor 647 (E-AB-F1392M), CXCR4-PE (E-AB-F1157D; all E-AB reagents from Elabscience, Wuhan, Hubei, China), PD-1-PE (EH12.2H7; BioLegend, San Diego, CA, USA), and an in-house FITC-conjugated anti-CD38 antibody. Secondary antibodies included goat anti-mouse Alexa Fluor 488 (A-11001) and 546 (A-11003), and goat anti-rabbit Alexa Fluor 488 (A-11008) and 546 (A-11010) (Thermo Fisher Scientific, Eugene, OR, USA).

Flow cytometric data were acquired on a CytoFLEX S instrument (Beckman Coulter, USA) equipped with four lasers (405, 488, 561, and 638 nm). Cell sorting was performed using a Sony MA900 system (Sony Biotechnology, San Jose, CA, USA). Data processing was conducted with CytExpert 2.0 software.

Secreted interferon-gamma (IFN-γ) and tumor necrosis factor-alpha (TNF) levels were quantified in culture supernatants collected 7 days post-lentiviral transduction using commercial ELISA kits (VECTOR-BEST, Novosibirsk, Russia). Absorbance was measured on a BIO-RAD iMark microplate reader (Bio-Rad Laboratories, Hercules, CA, USA).

### 2.8. HIV Co-Culture Infections

Single-round HIV infection assays were performed using an intron-regulated luciferase reporter system (inLuc), as previously described in articles [19,25,26,27]. Briefly, 1 × 10^6^ Raji cells were electroporated with the Neon Transfection System (1350 V, 30 ms, single pulse) using 2 μg pCMVΔ8.2R (Addgene #12263) packaging plasmid, 3 μg pUCHR-inLuc-mR reporter plasmid, and 0.8 μg of one of the following HIV envelope expression vectors: pIIINL4env (gift from Eric Freed, NCI-Frederick, NCI-Frederick, Frederick, MD, USA), pJRFLenv cells [28], or pZM135 (gift from E. Hunter and J. Blackwell, Emory University, GA, USA). Four to six h post-electroporation, transfected Raji cells were mixed at a 1:1 ratio with either unmodified CEM/R5 cells or CEM/R5 cells stably expressing one of the protective constructs, and cultured in 5 mL complete medium for 48 h. For primary cell infection assays, transfected Raji cells were co-cultured with freshly isolated CD4^+^ T lymphocytes at a 3:1 ratio for 72 h. Sixteen to eighteen h prior to harvest, 20 nM phorbol 12-myristate 13-acetate (PMA) was added to all co-cultures to enhance reporter gene expression in infected cells. Viral infectivity was quantified by measuring luciferase activity using GLO Lysis Buffer and the Bright-GLO Luciferase Assay System (Promega, Madison, WI, USA), with readings obtained on a GloMax-Multi Jr Multimode Reader (Promega, Madison, WI, USA).

### 2.9. HIV Spreading Assay

Viral spreading kinetics were assessed as previously described [19]. Infectious stocks of NL4-3, NL4-3mut, and NL(AD8) were generated by transfecting HEK293T cells with the corresponding molecular clones. Inoculum titers were standardized based on p24 antigen concentration. TCID_50_ (tissue culture infectious dose 50%) denotes the amount of virus required to produce detectable infection in 50% of target cultures. Briefly, 1 × 10^6^ CEM/R5 cells were infected with a high-dose inoculum (100 ng p24, approximately 400 TCID_50_) in 200 μL of culture medium for five h. Following incubation, cells were washed three times to remove residual virus and resuspended in 1 mL of complete growth medium. Partial medium exchange was performed every 48–72 h to maintain cell viability. Replication dynamics in model cell lines were monitored for 34 days. Primary CD4^+^ T lymphocytes were co-infected with a mixture of all three viral strains and monitored for 15 days. At designated time points, cells from both systems were harvested and analyzed for surface expression of the MT-C34 peptide and intracellular accumulation of HIV p24.

### 2.10. Chemotaxis Assay

CEM/R5 cell migration was evaluated following established protocols [19]. Briefly, 3 × 10^5^ cells were suspended in 100 μL of migration medium (RPMI supplemented with 4 mM glutamine, 10 μg/mL gentamicin, 0.3% BSA, and 10 mM HEPES, pH 7.2–7.4) and loaded into the upper compartment of 24-well Transwell inserts (8 μm pore size; SPL, TCS020024 Biofil, Guangzhou, China). The lower wells were pre-filled with 500 μL of the same medium supplemented with either 100 ng/mL SDF-1α (Abcam, Cambridge, UK) or control without SDF-1α. The plates were incubated for 6 h at 37 °C in a humidified atmosphere containing 5% CO2. Following incubation, migratory cells were harvested from the lower chamber and enumerated using an RWD C-100-SE automated cell counter (RWD Life Science, Shenzhen, China). Chemotactic activity was quantified as the percentage of cells migrated through the membrane relative to the total number of seeded cells.

### 2.11. Construction and Functional Validation of Doxycycline-Inducible Lentiviral Vectors

An inducible expression system was engineered using the Tet-On 3G platform (Takara Bio, Inc., Mountain View, CA, USA; Cat. No. 631168). The lentiviral transfer plasmid was designed such that the *CXCR4* coding sequence (or a *2P23-CXCR4* fusion variant) was placed under the control of a tetracycline-responsive promoter. The protective MT-C34 cassette and the reverse tetracycline transactivator (rtTA) were driven by constitutive human elongation factor-1 alpha promoter (EF1α). Promoters were oriented unidirectionally. Stable CEM/R5 cell lines were generated by lentiviral transduction followed by FACS for constitutive MT-C34 expression. To assess inducibility, sorted cells were cultured in complete medium supplemented with 1000 ng/mL doxycycline. Surface expression levels of CXCR4 and MT-C34 were quantified by flow cytometry at baseline and 24 h post-induction. Reversibility of the system was evaluated by removing doxycycline from the culture medium and monitoring transgene downregulation over 72 h. Chemotactic activity toward SDF-1 was assessed using Transwell migration assays in both uninduced and doxycycline-induced conditions. Antiviral efficacy was evaluated using cell-to-cell HIV transmission assays, comparing infection rates between uninduced and doxycycline-treated cultures.

### 2.12. Statistical Analysis

Experiments were conducted with 3–4 biological replicates. Data are reported as mean ± standard deviation. Comparisons between two independent groups were conducted using an unpaired, two-tailed Student’s *t*-test. Statistically significant differences were defined at *p* < 0.05.

## 3. Results

### 3.1. Bicistronic Lentiviral Architecture Enhances Surface Expression of Anti-HIV Peptides

Lentiviral transduction remains the most widely validated and clinically scalable platform for stable genomic delivery of therapeutic cassettes [29]. We hypothesized that a bicistronic vector architecture would substantially increase the surface density of membrane-associated viral entry inhibitors, thereby directly determining antiviral protection. Specifically, we proposed that co-expressing either two identical GPI-anchored C-peptides or two distinct inhibitory sequences under a single promoter would enhance cell-surface presentation and confer robust resistance to HIV infection.

**Figure 1:**
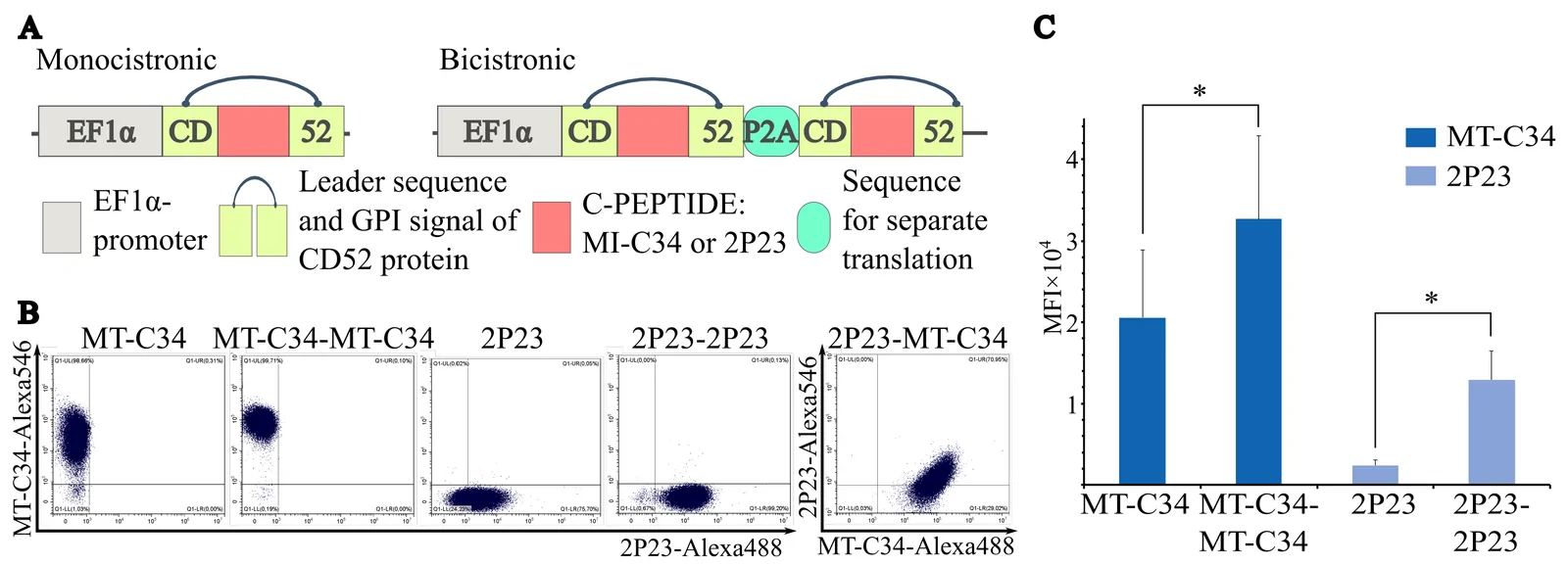
Development and characterization of therapeutic lentiviral vectors. (**A**) Schematic diagrams of the protective genetic construct of a monocistronic (**left**) and bicistronic (**right**) genetic cassette. The constructs include the EF1α promoter, signal sequences for export and GPI anchoring derived from the human CD52 protein, and a P2A sequence to ensure separate translation of two proteins from a single promoter; (**B**) Flow cytometry analysis of model cell lines CEM/R5 expressing the target genetic sequence. The level of surface expression of C-peptides was visualized using immunofluorescence staining and detected by flow cytometry; (**C**) Comparative analysis of the mean fluorescence intensity (MFI) values obtained after introducing protective genetic constructs using monocistronic and bicistronic vectors. The data were obtained from three independent experiments and are presented as mean values ± standard deviation; mean values were compared by two-tailed Student’s *t*-test, * *p* < 0.05.

To test this hypothesis, we engineered five lentiviral transfer plasmids: pCDH-MT-C34, pCDH-MT-C34-MT-C34, pCDH-2P23, pCDH-2P23-2P23, and pCDH-2P23-MT-C34 (Figure 1A). All therapeutic cassettes were driven by EF1α promoter, a constitutive non-viral promoter widely utilized in clinical gene therapy vectors for its high transcriptional activity [30]. The pCDH backbone incorporates a self-inactivating (SIN) 3′ long terminal repeat (LTR) to minimize insertional mutagenesis risk [29]. In bicistronic constructs, two protective elements were placed under a single EF1α promoter and separated by a porcine teschovirus-1 P2A self-cleaving peptide. Among the 2A family, P2A exhibits the highest ribosomal skipping efficiency, enabling stoichiometric production of discrete proteins from a single open reading frame [31]. Monocistronic variants contained a single copy of each therapeutic sequence.

CD52 was selected as the minimal GPI-anchored scaffold because it provides the optimal balance between compact size and robust surface expression among all tested GPI proteins. As a native human protein, CD52 is not expected to add immunogenicity. The C-peptide was substituted for the central region of CD52, between the N-glycosylation site and the GPI-attachment signal; substitution at this position preserves efficient export through the secretory pathway and membrane anchoring while displaying the inhibitory peptide in a conformation accessible for engagement with gp41 [27].

Third-generation lentiviral particles were generated via transient transfection of HEK293T cells using a four-plasmid packaging system, which eliminates the possibility of generating replication-competent lentivirus [29]. Viruses were pseudotyped with vesicular stomatitis virus glycoprotein (VSV-G). Payload modifications did not significantly affect viral titers (Supplementary Figure S2). Replication competence was rigorously excluded by culturing transduced HEK293T cells for 30 days and confirming undetectable p24 antigen levels in culture supernatants via ELISA.

Stable cell lines were established by transducing the HIV-permissive cell line CEM/R5 at a low MOI, followed by FACS. Genomic integration levels were quantified using primers specific to the WPRE cassette and the comparative ΔΔCt method [32] and showed no significant differences between monocistronic and corresponding bicistronic pairs (MT-C34 vs. MT-C34-MT-C34; 2P23 vs. 2P23-2P23) (Supplementary Figure S3). Flow cytometric analysis demonstrated that bicistronic vectors significantly increased surface expression of anti-HIV peptides relative to their monocistronic counterparts (*p* < 0.05) (Figure 1B,C). Furthermore, the bicistronic construct containing different protective elements enabled the simultaneous expression of two different protective peptides (Figure 1B, right cytogram).

### 3.2. Bicistronic Lentiviral Vectors Confer Enhanced Protection Against HIV Infection in Cell Lines

**Figure 2:**
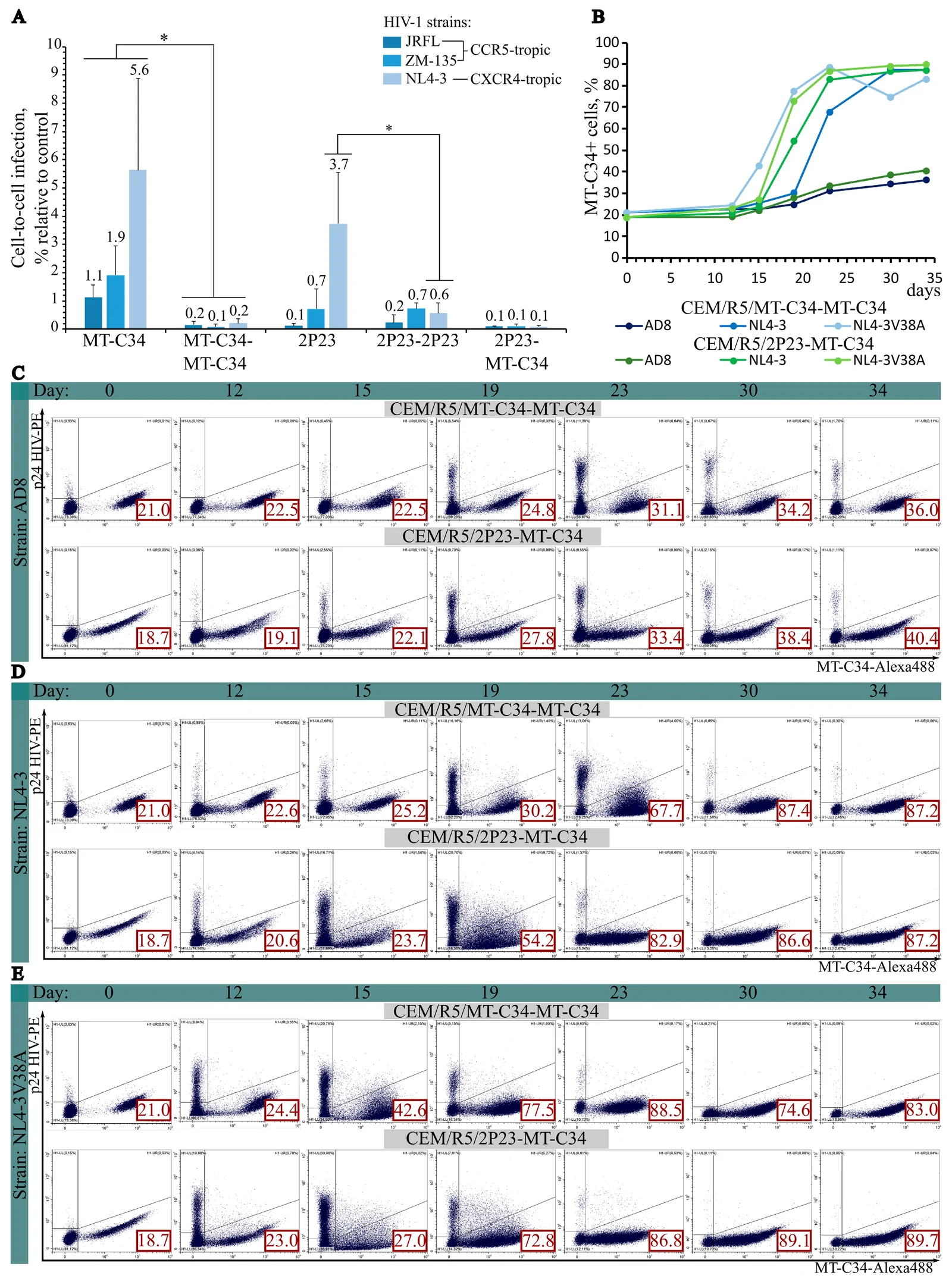
Infection assays with model cell lines harboring the protective constructs. (**A**) Normalized level of HIV cell-to-cell transmission. The data from infectious tests for the control cell line CEM/R5 are taken as 100%. Infection assays were performed with pseudoviruses carrying envelopes with distinct coreceptor tropisms: the CCR5-tropic JRFL and ZM-135 and the CXCR4-tropic NL4-3. Data obtained from at least three independent experiments are presented as mean values ± standard deviation. Mean values were compared by two-tailed Student’s *t*-test, * *p* < 0.05; (**B**) Kinetics of MT-C34^+^ cell enrichment over 34 days post-infection for HIV-1 strains AD8, NL4-3, and NL4-3V38A. Blue and green color families indicate two independent cell lines (CEM/R5/MT-C34-MT-C34 and CEM/R5/2P23-MT-C34, respectively) (**C**–**E**). Infection assays with replication-competent HIV strains. A mixture of cells comprising approximately 80% CEM/R5 cells and 20% cells from the same line transduced with protective constructs MT-C34-MT-C34 (upper panels) or 2P23-MT-C34 (lower panels) were infected with a replication-competent HIV strain at ~400 TCID_50_: (**B**) CCR5-tropic strain AD8, (**C**) CXCR4-tropic strain NL4-3, (**D**) CXCR4-tropic strain NL4-3mut38, which carries a mutation in the envelope responsible for the development of enfuvirtide resistance. The red boxes show the percentage of MT-C34^+^ cells. In all tests presented, following the peak of infection, there was an increase in the proportion of cells harboring the protective construct and a decrease in the number of non-transduced cells. The greatest reduction in infection level and the least enrichment of the cell population with the peptide were observed with the AD8 strain, presumably due to the low level of CCR5 expression on the cell surface (Supplementary Figure S4).

The protective efficacy of the engineered lentiviral vectors was evaluated using intercellular transmission assays with HIV pseudoviruses of distinct tropisms. Viral entry was quantified using an inLuc vector [19], in which reporter expression is strictly coupled to completion of the viral replication cycle. The inclusion of an intron and an inverted expression cassette ensures that luminescence is detected exclusively in successfully infected target cells, even when producer and recipient populations are co-cultured. Comparison of monocistronic and bicistronic constructs revealed superior protection conferred by the latter (Figure 2A). Specifically, cells harboring the MT-C34-MT-C34 construct exhibited significantly enhanced resistance across all tested viral tropisms, whereas the 2P23-2P23 construct provided markedly improved protection against the CXCR4-tropic strain NL4-3. Co-expression of two surface-anchored peptides consistently yielded robust, tropism-independent antiviral activity.

Among all variants, the MT-C34-MT-C34 and 2P23-MT-C34 constructs demonstrated the highest efficacy, achieving 99.8–99.9% cellular protection. These lines were subsequently challenged with replication-competent HIV strains: the CCR5-tropic AD8, the CXCR4-tropic NL4-3, and NL4-3V38A, which carries a V38A envelope mutation conferring enfuvirtide resistance (Figure 2C–E). Following inoculation, cultures were maintained in complete medium with partial passaging every 48–72 h as described in the Section 2. Over a 34-day observation period, both constructs sustained potent antiviral activity. Intracellular p24 accumulation was restricted exclusively to peptide-negative cells. While control cultures succumbed to cytopathicity following the infection peak, transduced cells remained viable and continued to proliferate. Specifically, in the lowest observed enrichment, the modified cell population expanded from 21% to 36%, whereas in the highest observed enrichment, it expanded from 18.7% to 89.7%. Consequently, the proportion of protected cells increased by 15 to 71 percentage points, depending on the viral strain and cell line. Overall enrichment dynamics are summarized in Figure 2B. Notably, replication-competent virus was completely eliminated from co-cultures before unmodified target cells were fully depleted. This phenomenon likely reflects a bystander neutralization effect: membrane-anchored C-peptides bind gp41 on virions attempting to infect adjacent unmodified cells, inducing irreversible conformational changes that abrogate viral infectivity.

Collectively, these data demonstrate that bicistronic lentiviral vectors confer durable, tropism-independent resistance to HIV entry. Under sustained viral pressure, genetically modified cells exhibit a pronounced selective survival advantage, maintaining viability and proliferative capacity while progressively outcompeting susceptible counterparts.

### 3.3. Bicistronic Lentiviral Vectors Confer Protection of Primary CD4^+^ T Lymphocytes Against HIV Infection

**Figure 3:**
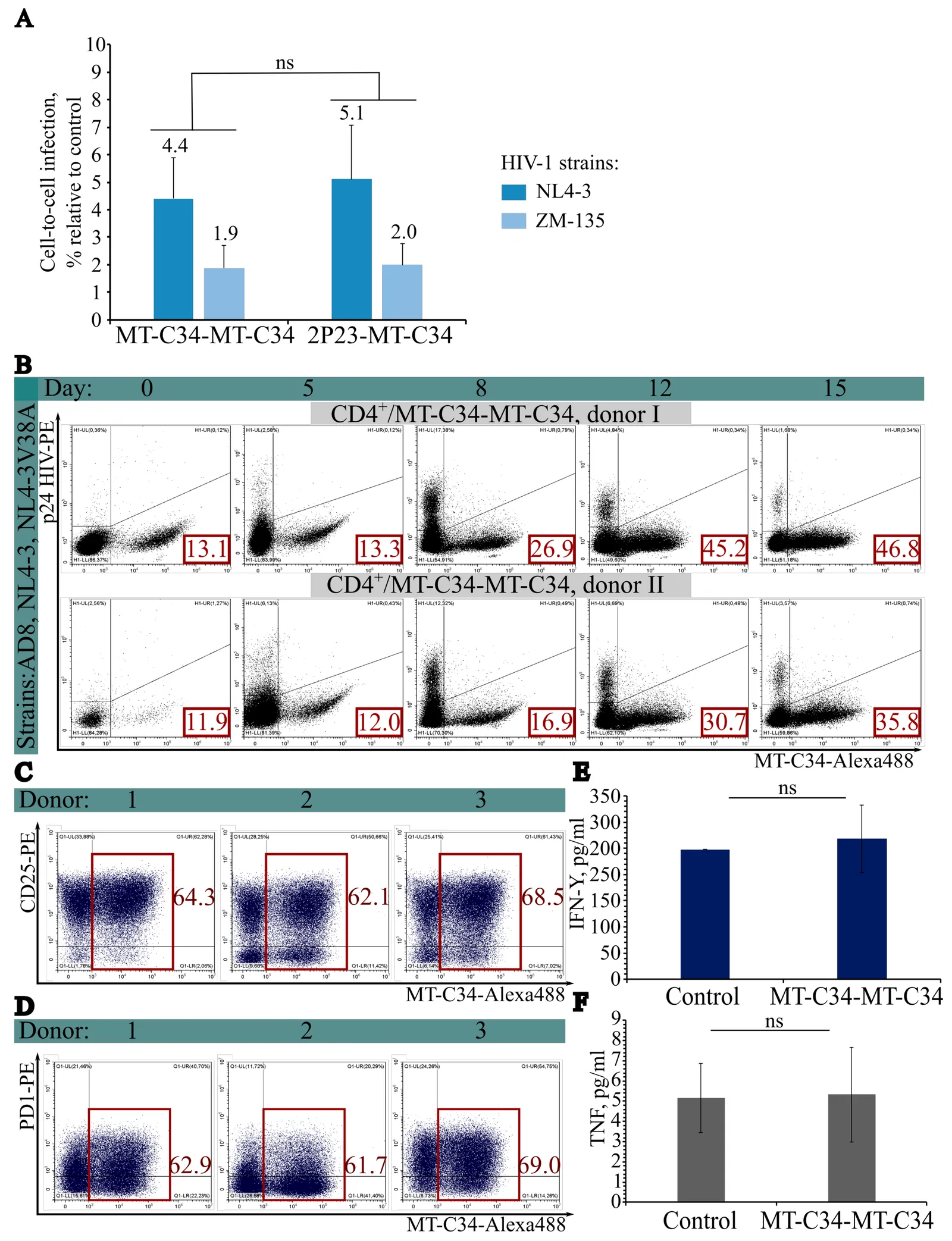
Protective activity and phenotypic effects of the developed bicistronic vectors on human primary CD4^+^ lymphocytes. (**A**) Resistance of modified lymphocytes to intercellular HIV transmission with different tropisms. CD4^+^ lymphocytes from three donors were transduced with lentiviral vectors and sorted. Following cell sorting, the proportion of modified cells within the population was evaluated, and infection assays were conducted to assess intercellular virus transmission. (**B**) Dynamics of resistance to infection with a mixture of replication-competent HIV strains, including a strain harboring an envelope mutation that confers resistance to the soluble fusion inhibitor T-20 (enfuvirtide). The red boxes show the percentage of MT-C34+ cells. (**C**,**D**) Flow cytometry analysis of surface expression levels of the activation and proliferation marker CD25 (**C**) and the exhaustion marker PD1 (**D**) in CD4^+^ lymphocytes; MT-C34^+^ and MT-C34^−^ populations are compared. (**E**) Analysis of IFN-γ content in culture supernatants of transduced and control CD4^+^ lymphocytes on day 5 post-transduction, performed by ELISA. (**F**) Comparison of TNF content in the supernatants of modified and control CD4^+^ lymphocytes on day 7 post-transduction, performed using the ELISA method. Mean values ± standard deviation are presented. Mean values were compared by two-tailed Student’s *t*-test (ns—not statistically significant).

We next evaluated the protective efficacy of the bicistronic lentiviral vectors pCDH-MT-C34-MT-C34 and pCDH-2P23-MT-C34 in primary human CD4^+^ T lymphocytes. Intercellular transmission assays were performed using HIV pseudoviruses with distinct tropisms (Figure 3A). Both constructs conferred robust protection against viral entry during cell-to-cell transfer. The MT-C34-MT-C34 construct yielded marginally higher transgene expression and slightly improved protection compared to 2P23-MT-C34; however, this difference did not reach statistical significance. Residual infectivity observed in sorted populations likely reflects transient functional masking of the protective peptide by anti-C-peptide antibodies used during FACS.

To assess protection against authentic virus, we conducted co-culture experiments using replication-competent HIV strains. CD4^+^ T cells from two donors were transduced with the MT-C34-MT-C34 vector. Following confirmation of transduction efficiency, modified cells were mixed with unmodified autologous cells at an approximate ratio of 10:90. Each mixture was inoculated simultaneously with three replication-competent strains—AD8, NL4-3, and NL4-3V38A at ~400 TCID_50_ of each strain (Figure 3B). Viral replication peaked on day 8, followed by a progressive decline. This reduction correlated with expansion of the MT-C34^+^ population and concomitant loss of MT-C34^−^ cells due to HIV-induced cytopathicity. By day 15, the proportion of protected cells increased by 37 percentage points (donor 1) and 28 percentage points (donor 2), accompanied by near-complete viral clearance in donor 1 and a clear downward trend in donor 2.

These findings demonstrate that bicistronic lentiviral vectors effectively shield primary CD4^+^ T cells from replication-competent HIV. The selective expansion of MT-C34^+^ cells under sustained viral pressure supports a competitive survival advantage for genetically modified lymphocytes.

To determine whether lentiviral modification altered cellular physiology, we analyzed activation, exhaustion, proliferation, and cytokine secretion profiles. CD4^+^ T cells from three donors were transduced with MT-C34-MT-C34 and stained for the activation marker CD25, the exhaustion marker PD-1, and surface MT-C34 (Figure 3C,D). Transduction efficiencies exceeded 60% across all donors, confirming protocol robustness. Expression levels of CD25 and PD-1 were comparable between MT-C34^+^ and MT-C34^−^ subsets, indicating that genetic modification does not perturb activation or exhaustion pathways. Proliferative capacity was assessed over 10 days post-transduction, yielding an average 19-fold expansion (Supplementary Figure S5).

A critical prerequisite for clinical translation is the absence of unintended vector-induced activation. To evaluate potential inflammatory responses, culture supernatants were analyzed by ELISA for IFN-γ and TNF. IFN-γ levels remained within baseline ranges following transduction, indicating no pronounced interferon response (Figure 3E). Similarly, TNF secretion showed no significant elevation (Figure 3F).

Collectively, these data indicate that the bicistronic lentiviral platform enables high-efficiency transduction of primary CD4^+^ T lymphocytes without compromising key physiological parameters. Modified cells retain normal activation kinetics, proliferative capacity, and homeostatic cytokine profiles. These findings support the translational potential of this approach for ex vivo gene therapy of HIV infection.

### 3.4. Bicistronic Lentiviral Vectors Enable Efficient Delivery of Protective Constructs into Human HSCs

Modern HIV therapies aim to achieve sustained viral suppression and prevent transmission. One promising strategy involves introducing therapeutic sequences into the genome of CD34^+^ HSCs. Allogeneic transplantation of HSCs from donors carrying the CCR5Δ32 mutation can eradicate CCR5-tropic HIV, as demonstrated in Berlin [2,3,4] and London [5,6] patients. However, this approach does not confer resistance against CXCR4-tropic strains, which may lead to viral rebound [1,8]. To address this limitation, we developed a strategy to introduce our therapeutic constructs directly into the HSC genome.

**Figure 4:**
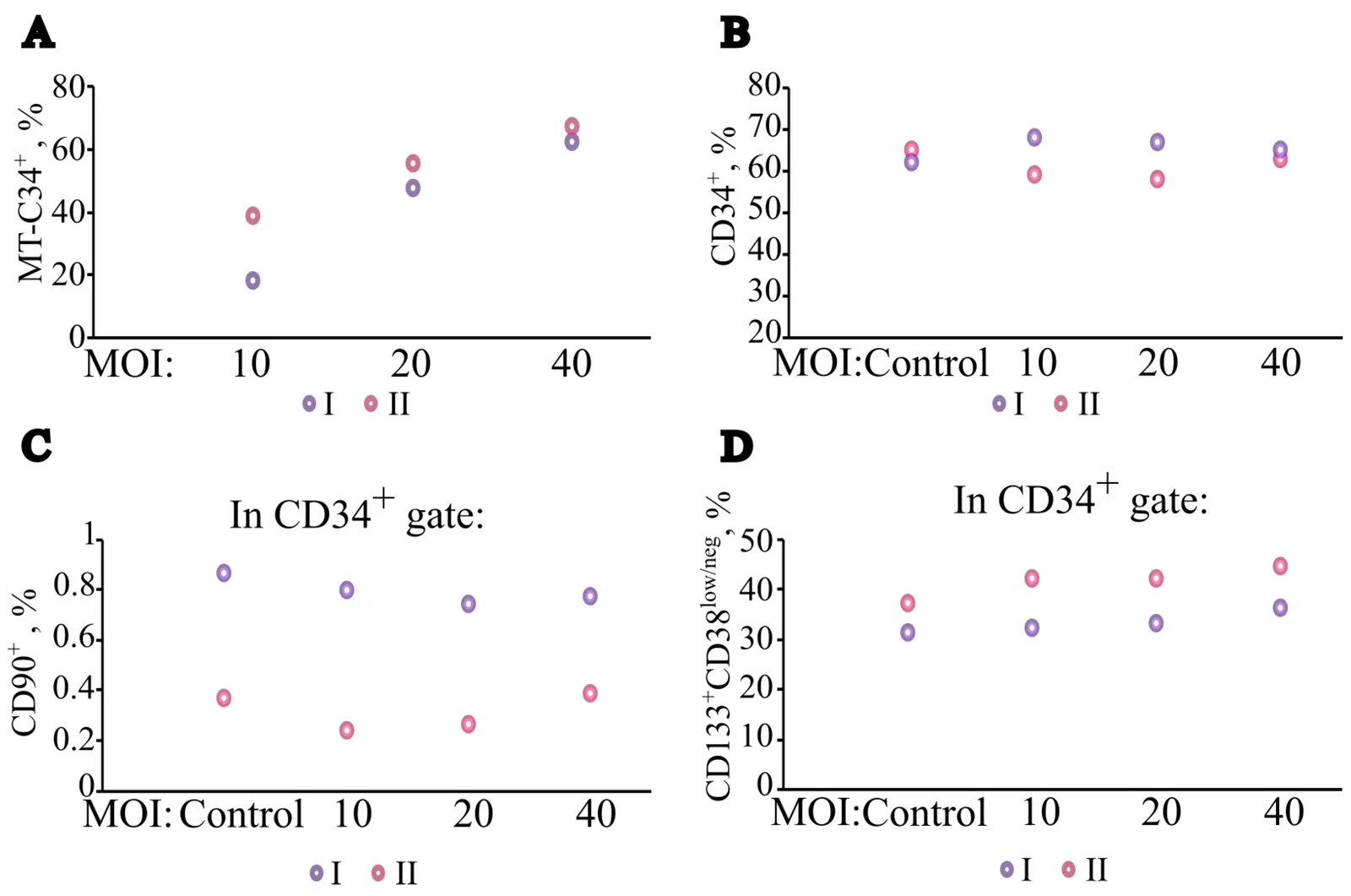
Analysis of lentiviral transduction efficiency and its impact on the expression of HSC stemness markers. (**A**) Efficiency of lentiviral transduction at different MOIs. (**B**) CD34^+^ cell content on day 6 after lentiviral transduction in the transduced and control samples. (**C**) CD90^+^ cell content in the transduced and control samples. (**D**) CD133^+^CD38^low/−^ cell content in the CD34^+^ HSCs population in transduced and control HSCs. All data are shown for two different donors.

We first optimized culture conditions to promote HSC expansion, testing various combinations of SCF, FL, TPO, and IL-6. Using HSCs from two healthy donors, we determined that maximal expansion and viability were achieved with 100 ng/mL SCF, 100 ng/mL FL, 10 ng/mL TPO, and 60 ng/mL IL-6 (Supplementary Figure S6). Next, we optimized transduction of the lentivirus with the therapeutic construct MT-C34-MT-C34 by testing MOI of 10, 20, and 40 (Figure 4A). Transduction efficiency increased dose-dependently: at MOI 10, efficiencies were 18% and 39% for donors 1 and 2, respectively; at MOI 20, 50% and 55%; and at MOI 40, 62% and 67%.

For clinical translation, preserving the engraftment capacity of modified HSCs is critical. HSCs characterized by the CD34^+^CD133^+^CD38^low/neg^CD90^+^ phenotype exhibit the highest repopulating potential [33,34]. Therefore, we evaluated whether lentiviral transduction altered these stemness markers (Supplementary Figure S7). Flow cytometric analysis revealed that transduction did not significantly affect the expression of CD34, CD90, CD133, or CD38 across all tested MOIs (Figure 4B–D). Although the CD90^+^ subset was small in both donors (~0.8% and ~0.3% of the CD34^+^ population, respectively; Figure 4C), its proportion remained unchanged post-transduction. These results demonstrate that our protocol achieves ≥60% transduction efficiency while preserving key stemness markers, supporting its potential for clinical application.

### 3.5. Optimization of Lentiviral Vector Architectures to Enhance Bone Marrow Homing of Genetically Modified HSCs

Allogeneic HSC transplantation remains a cornerstone therapy for hematologic and immunologic disorders; however, myeloablative conditioning regimens carry significant morbidity and mortality. Consequently, developing conditioning-independent approaches that promote the engraftment of genetically modified HSCs within the bone marrow niche represents a critical unmet clinical need. One promising strategy involves enhancing the chemotactic homing of modified HSCs through *CXCR4* overexpression, which directs cells toward stromal cell-derived factor-1 (SDF-1/CXCL12) gradients in the marrow [35]. CXCR4 is a pivotal chemokine receptor governing HSC trafficking, and its modulation has been shown to improve homing efficiency and hematopoietic recovery [36,37,38]. However, constitutive *CXCR4* upregulation presents a fundamental challenge: CXCR4 serves as a coreceptor for HIV entry. Given that HSCs express CD4 and are susceptible to HIV infection [39,40], a viable therapeutic strategy must simultaneously enhance homing and confer robust antiviral protection. We engineered lentiviral vectors that co-express CXCR4 with HIV-protective C-peptides and developed an inducible expression system to enable transient, controlled *CXCR4* upregulation, thereby mitigating the risk of the WHIM syndrome associated with sustained *CXCR4* hyperactivation [41].

**Figure 5:**
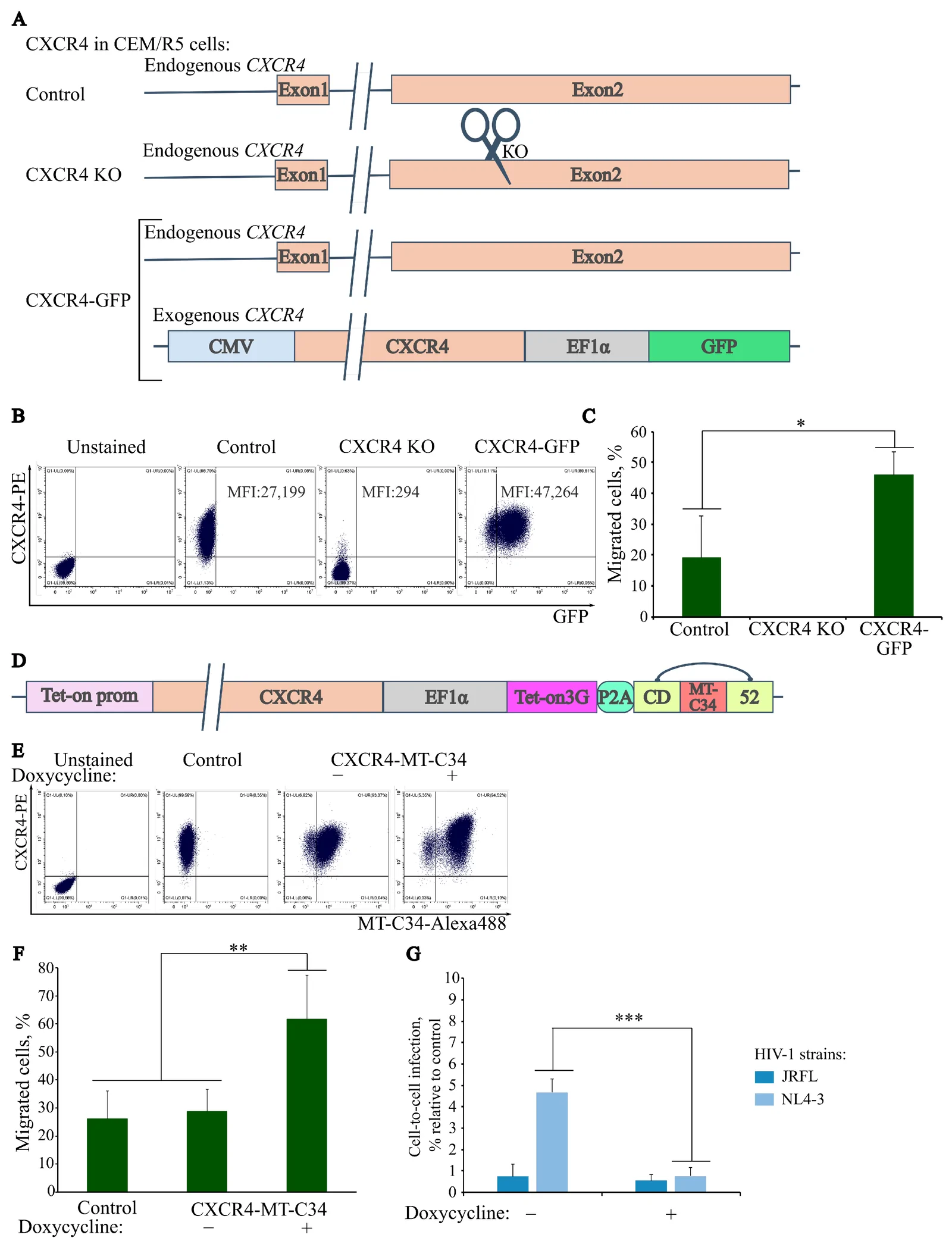
Development of a therapeutic lentiviral vector sequence that simultaneously enhances the chemotactic activity of modified cells and protects them from HIV infection. (**A**) Schemes of CXCR4 configurations in model cell lines. (**B**) Flow cytometry analysis of CXCR4 expression in the model cell lines. The cytofluorograms indicate the MFI of the CXCR4^+^ population. (**C**) Chemotaxis activity level toward SDF-1 of CEM/R5 cell lines carrying different combinations of CXCR4. (**D**) Schematic representation of a lentiviral vector providing inducible expression of CXCR4 (via the Tet-On promoter) and constitutive expression of the protective MT-C34 construct. The Tet-On promoter is activated by the Tet-On 3G transactivator sequence in the presence of doxycycline. (**E**) Flow cytometry analysis of the expression level of the protective construct and CXCR4 depending on the addition of doxycycline. (**F**) Chemotaxis activity level toward SDF-1-derived model cell lines and controls depending on the presence of doxycycline in the medium. (**G**) Protective activity of the developed constructs, investigated in tests on the intercellular transmission of HIV with different tropisms. Mean values ± standard deviation are presented. Mean values were compared by two-tailed Student’s *t*-test, * *p* < 0.05, ** *p* < 0.01, *** *p* < 0.001.

To establish a functional baseline for CXCR4-mediated homing, we generated a lentiviral vector driving *CXCR4* and the fluorescent reporter GFP (Figure 5A). Following transduction of the CEM/R5 cell line and FACS, we established the CEM/R5/CXCR4-GFP model. In parallel, CRISPR/Cas9-mediated knockout of endogenous *CXCR4*, combined with cell sorting, yielded a CXCR4-deficient line. Flow cytometric analysis confirmed complete ablation of surface CXCR4 in the knockout line, whereas exogenous expression increased MFI of surface CXCR4 by approximately 1.7-fold relative to untransduced controls (Figure 5B). Directed migration assays in response to SDF-1 demonstrated that *CXCR4*-knockout cells were completely non-responsive, whereas cells expressing exogenous CXCR4 exhibited significantly enhanced chemotactic migration (Figure 5C).

While N-terminally fused C-peptides can prevent CXCR4-mediated HIV entry, their application is often limited. For instance, the N-terminal fusion of the C34 peptide has been shown to impair CXCR4 chemotactic activity [38]. To address this limitation, we investigated whether the shorter, highly active anti-HIV C-peptide 2P23 [19,21] could antagonize viral coreceptor function without compromising receptor-mediated migration. We constructed two lentiviral vectors enabling co-expression of CXCR4 with 2P23 and GFP under the CMV promoter (CMV-2P23-CXCR4-GFP), and CXCR4 with 2P23 combined with the GPI-anchored peptide MT-C34 under the EF1α promoter (EF1α-2P23-CXCR4-MT-C34) (Supplementary Figure S8A). Flow cytometry verified stable upregulation of surface CXCR4 and concurrent membrane localization of the delivered peptides (Supplementary Figure S8B,C). Notably, 2P23 did not impair chemotactic activity; instead, migration was significantly enhanced in both engineered lines, with the EF1α promoter exhibiting a stronger chemotactic response (Supplementary Figure S8D). In cell-to-cell HIV transmission assays, both lines exhibited complete resistance to infection by both R5- and X4-tropic viral strains (Supplementary Figure S8E). Furthermore, transduction of primary CD4^+^ T cells from two healthy donors with EF1α-2P23-CXCR4-MT-C34 significantly enhanced their SDF-1-directed chemotaxis (Supplementary Figure S8F,G).

Given that sustained *CXCR4* hyperactivation predisposes to WHIM syndrome [41], we developed an inducible Tet-On 3G-based expression system to enable transient, controlled *CXCR4* upregulation while maintaining constitutive antiviral protection. In this architecture, *CXCR4* (or a *2P23–CXCR4* fusion) was placed under a tetracycline-responsive promoter, whereas the MT-C34 protective cassette and the tetracycline transactivator were driven by the constitutive EF1α promoter and separated by a P2A self-cleaving sequence (Figure 5D). Both promoters were oriented unidirectionally to enable read-through transcription upon doxycycline induction, thereby ensuring transient expression of the anti-HIV protective construct alongside CXCR4 to compensate for the heightened risk of viral entry during receptor overexpression. Using this construct, we generated the CEM/R5/CXCR4-MT-C34 cell line. Flow cytometric profiling revealed baseline CXCR4 levels comparable to controls, with constitutive MT-C34 expression. Upon addition of doxycycline, surface expression of both CXCR4 and MT-C34 increased 24 h later (Figure 5E). Consistently, the CXCR4-MT-C34 construct exhibited tightly regulated migration: baseline chemotaxis matched uninduced controls, whereas doxycycline-mediated induction triggered a robust, statistically significant increase in SDF-1-directed migration (Figure 5F).

To evaluate the reversibility of doxycycline-mediated CXCR4 induction, we engineered the CEM/R5/2P23-CXCR4-MT-C34 cell line expressing the 2P23-CXCR4 fusion construct, in which the 2P23 peptide serves as a convenient reporter for tracking induction dynamics (Supplementary Figure S9A). Removal of doxycycline resulted in complete restoration of CXCR4 and MT-C34 levels to pre-induction baselines within 72 h (Supplementary Figure S9F). Interestingly, the 2P23-CXCR4 fusion failed to produce a statistically significant increase in surface CXCR4 density upon induction, despite detectable 2P23 at the plasma membrane (Supplementary Figure S9B,D). At the same time, the MFI value of MT-C34 increased after the addition of doxycycline, which indicates the correct functioning of the expression cassette (Supplementary Figure S9C). This discrepancy may stem from altered intracellular trafficking, steric interference with receptor maturation, or competitive inhibition with endogenous CXCR4 pools, ultimately limiting stable surface retention of the fusion protein. Consistently, the chemotactic response in this fusion-expressing line was variable and statistically indistinguishable from controls (Supplementary Figure S9E).

Antiviral efficacy was further assessed using cell-to-cell HIV transmission assays. The inducible CEM/R5/CXCR4-MT-C34 line maintained a high level of protection against HIV infection, and doxycycline-mediated induction significantly enhanced resistance to X4-tropic virus, consistent with the transient upregulation of MT-C34 via read-through transcription (Figure 5G and Figure S9C).

## 4. Discussion

In this study, we demonstrate that a bicistronic lentiviral architecture substantially increases the surface density of membrane-associated viral entry inhibitors, proving that expression levels are a primary determinant of antiviral potency. Our data demonstrate that simultaneous expression of two identical or distinct C-peptides separated by a P2A self-cleaving peptide and expressed under the control of the EF1α promoter surpasses the limitations of monocistronic constructs. Ultimately, this design achieves robust surface expression and confers broad, tropism-independent protection against HIV.

These findings align with current paradigms in cellular therapy, wherein clinical efficacy of membrane-expressed molecules correlates strictly with their surface density. For example, long-term therapeutic responses in CAR-T cell therapy correlate with chimeric antigen receptor expression levels [37]. Similarly, for fusion-inhibiting peptides, increased surface density enhances antiviral protection, whereas sparse expression compromises efficacy [19].

We showed that replication-competent HIV was completely eliminated from co-cultures before unmodified target cells were fully depleted. We propose that this phenomenon may be driven by a bystander neutralization effect: membrane-anchored C-peptides bind gp41 on virions attempting to infect neighboring unmodified cells, inducing conformational changes that render the viral particles non-infectious. This mechanism appears to block cell-to-cell transmission and establish a localized protective barrier. From a translational perspective, sustained viral suppression may be achievable at relatively low doses of therapeutically modified cells; however, this hypothesis requires validation in vivo.

We acknowledge that the spatial distribution of GPI-anchored molecules may differ between cell lines and migrating primary lymphocytes, where such proteins often accumulate at the uropod rather than at the leading edge, where chemokine receptors actively scan the extracellular environment. However, recent evidence indicates that chemokine receptor distribution on migrating T cells is not fixed but highly dynamic and condition-dependent: during constitutive migration, receptor distribution is largely isotropic, and only upon chemokine exposure does it exhibit a transient bias toward the uropod before progressively redistributing across the cell surface [42]. Such plasticity further complicates the assumption that precise subcellular positioning per se determines antiviral efficacy. Our prior work directly tested this assumption by asking whether forcing the fusion inhibitor into close proximity with HIV coreceptors enhances antiviral potency. Specifically, we engineered a construct (MT-C34-15D) in which the C-peptide was non-covalently tethered to HIV coreceptors via the 15D peptide derived from gp120, thereby forcing the inhibitor into close proximity with CXCR4 and CCR5. Contrary to expectations, this modification did not improve—and sometimes reduced—the protective activity of MT-C34 relative to the standard GPI-anchored form, which is expressed separately in lipid rafts. These data indicate that physical recruitment of the fusion inhibitor to the coreceptor site does not confer additional benefit, suggesting that the protective capacity of the construct is governed primarily by its surface density and accessibility at any site of membrane apposition rather than by precise sub-cellular positioning [19].

Importantly, co-expression of two structurally distinct C-peptides establishes a dual-barrier architecture that substantially raises the mutational threshold required for viral escape. Because envelope mutations conferring resistance to one inhibitor typically do not compromise binding to the other, HIV must simultaneously acquire non-overlapping gp41 modifications to overcome protection—a markedly higher genetic burden than that imposed by any single-peptide construct. This principle parallels the clinical rationale underlying combination antiretroviral therapy and is expected to delay the emergence of resistant variants, including those selected under pressure from soluble fusion inhibitors such as enfuvirtide.

Several limitations must be acknowledged when interpreting these results. All experiments were conducted in vitro using model cell lines and primary cells from a limited number of donors. Immunogenicity of heterologous peptides, long-term stability, and potential impacts on the bone marrow microenvironment require rigorous validation in animal models. It must also be verified that the modifications do not impair cellular capacity for self-renewal and differentiation. The extent to which this platform offers advantages over existing systems remains to be determined experimentally.

The lentiviral vector developed in this study incorporates a therapeutic module containing *CXCR4* under the control of a Tet-On system. Transduced cells exhibited enhanced chemotaxis toward SDF-1, indicating potential support for HSC homing. The inducible promoter should restrict *CXCR4* upregulation to the engraftment phase, potentially mitigating risks associated with sustained *CXCR4* overexpression. This strategy may advance HIV gene therapy by enabling enrichment of the genetically modified HSCs niche while eliminating the requirement for intensive conditioning regimens. It has been shown that spontaneous mutations leading to gene mosaicism, where a mutation causes primary immunodeficiency, can result in a milder course [37,43]. Such cases demonstrate that complete replacement of endogenous HSCs with modified cells is not always necessary; niche enrichment alone can yield significant clinical benefits. Overall, the platform developed in our study can be adapted for treating a broad spectrum of monogenic diseases.

The bicistronic lentiviral architecture described here may have applications in other modalities where per-cell expression levels are limiting, such as CAR-T therapy [40] or delivery of neurotrophic factors including GDNF [44] and BDNF [45]. Long-term follow-up of 38 patients receiving CAR-T therapy for B-cell lymphoma demonstrated that overall survival depends on CAR expression levels during the first two years post-infusion, suggesting that amplification of per-cell CAR production could be particularly beneficial in this setting [37]. Similarly, delivery of small neurotrophic factors such as GDNF and BDNF to the central nervous system may require supraphysiological secretion levels to overcome diffusion barriers—a limitation that increased per-cell expression could potentially address. Although GDNF and BDNF are delivered via AAV vectors in published studies, this does not preclude adaptation to our platform, as the genetic sequences for GDNF and BDNF are short; duplication within the vector backbone would not exceed packaging capacity.

Given the modular design of the bicistronic lentiviral platform and its compatibility with established GMP manufacturing pipelines, it holds measurable translational potential for advancing both infectious disease therapies and cellular engineering strategies, contingent upon successful preclinical validation.

## 5. Conclusions

In this study, we developed bicistronic lentiviral vectors to engineer primary CD4^+^ T cells and HSCs for the surface expression of anti-HIV C-peptides. The modified cells maintained >99.8% protection against HIV infection without detectable impairment of physiological functions. An inducible CXCR4-based system was included to regulate homing-related signaling. The correlation between increased surface protein density and enhanced protective efficacy suggests that this design principle may be applicable to other cell-based therapies. Preclinical validation in relevant animal models will be necessary to determine whether this platform is suitable for clinical applications.

## 6. Patents

The technology described herein is covered by Russian Federation Patents No. 2857827 and No. 2830859, assigned to IBG RAS.

## Data Availability

The original contributions presented in this study are included in the article/Supplementary Materials. Further inquiries can be directed to the corresponding author.

## Supplementary Materials

The following supporting information can be downloaded at: https://www.mdpi.com/article/10.3390/cells15181704/s1, Figure S1: Alignment scheme of C-peptides T20, 2P23, and MT-C34 to the HIV-1 gp41 sequence; Figure S2: Viral titers produced with lentivectors carrying different gene therapy constructs; Figure S3: The integration level was estimated by Delta Delta (ΔΔCt) Method for Relative Quantification and was equal in cell lines with 2P23 and 2P23-2P23 constructs and with MT-C34 and MT-C34-MT-C34 constructs; Figure S4: Quantification of CXCR4 and CCR5 surface expression levels in CEM/R5 cells via flow cytometry.; Figure S5: Dynamics of CD4^+^ lymphocyte growth; Figure S6: Selection of the optimal ratio of cytokines in the culture medium in an experiment with HSCs from two donors; Figure S7: Analysis of the efficiency of lentiviral transduction and its impact on the expression of hematopoietic stemness markers; Figure S8: Functional characterization of lentivirally delivered CXCR4–2P23 fusion constructs; Figure S9: Functional characterization of the doxycycline-inducible 2P23–CXCR4 lentiviral construct; Table S1: Description of the gene therapy sequences; Table S2: qPCR primer sequences.

## Abbreviations

The following abbreviations are used in this manuscript:

CEM/R5	T-lymphoblastic cell line CCRF-CEM/R5
FBS	Fetal bovine serum
FACS	Fluorescence-activated cell sorting
FL	Flt3 ligand
EF1α	Human elongation factor-1 alpha
HIV	Human immunodeficiency virus type 1
HSCs	Hematopoietic stem cells
IFN-γ	Interferon-gamma
IL-6	Interleukin-6
inLuc	Intron-regulated luciferase reporter
MFI	Mean fluorescence intensity
MOI	Multiplicity of infection
PBMCs	Peripheral blood mononuclear cells
qPCR	Real-Time PCR
SCF	Stem cell factor
TCID_50_	Tissue culture infectious dose 50%
TNF	Tumor necrosis factor
TPO	Recombinant human thrombopoietin
VSV-G	Vesicular stomatitis virus glycoprotein
WHIM syndrome	Warts, Hypogammaglobulinemia, Infections, and Myelokathexis syndrome
WPRE	Woodchuck hepatitis virus post-transcriptional regulatory element

## References

[1] Maslennikova A., Mazurov D. (2022). Application of CRISPR/Cas Genomic Editing Tools for HIV Therapy: Toward Precise Modifications and Multilevel Protection. Front. Cell. Infect. Microbiol..

[2] Hütter G., Nowak D., Mossner M., Ganepola S., Müßig A., Allers K., Schneider T., Hofmann J., Kücherer C., Blau O. (2009). Long-Term Control of HIV by CCR5 Delta32/Delta32 Stem-Cell Transplantation. N. Engl. J. Med..

[3] Allers K., Hütter G., Hofmann J., Loddenkemper C., Rieger K., Thiel E., Schneider T. (2011). Evidence for the Cure of HIV Infection by CCR5Δ32/Δ32 Stem Cell Transplantation. Blood.

[4] Yukl S.A., Boritz E., Busch M., Bentsen C., Chun T.-W., Douek D., Eisele E., Haase A., Ho Y.-C., Hütter G. (2013). Challenges in Detecting HIV Persistence during Potentially Curative Interventions: A Study of the Berlin Patient. PLoS Pathog..

[5] Gupta R.K., Abdul-Jawad S., McCoy L.E., Mok H.P., Peppa D., Salgado M., Martinez-Picado J., Nijhuis M., Wensing A.M.J., Lee H. (2019). HIV-1 Remission Following CCR5Δ32/Δ32 Haematopoietic Stem-Cell Transplantation. Nature.

[6] Gupta R.K., Peppa D., Hill A.L., Gálvez C., Salgado M., Pace M., McCoy L.E., Griffith S.A., Thornhill J., Alrubayyi A. (2020). Evidence for HIV-1 Cure after CCR5Δ32/Δ32 Allogeneic Haemopoietic Stem-Cell Transplantation 30 Months Post Analytical Treatment Interruption: A Case Report. Lancet HIV.

[7] Mock U., Machowicz R., Hauber I., Horn S., Abramowski P., Berdien B., Hauber J., Fehse B. (2015). MRNA Transfection of a Novel TAL Effector Nuclease (TALEN) Facilitates Efficient Knockout of HIV Co-Receptor CCR5. Nucleic Acids Res..

[8] Kordelas L., Verheyen J., Esser S. (2014). Shift of HIV Tropism in Stem-Cell Transplantation with CCR5 Delta32 Mutation. N. Engl. J. Med..

[9] Yu S., Yao Y., Xiao H., Li J., Liu Q., Yang Y., Adah D., Lu J., Zhao S., Qin L. (2017). Simultaneous Knockout of CXCR4 and CCR5 Genes in CD4^+^ T Cells via CRISPR/Cas9 Confers Resistance to Both X4- and R5-Tropic Human Immunodeficiency Virus Type 1 Infection. Hum. Gene Ther..

[10] Liu Z., Chen S., Jin X., Wang Q., Yang K., Li C., Xiao Q., Hou P., Liu S., Wu S. (2017). Genome Editing of the HIV Co-Receptors CCR5 and CXCR4 by CRISPR-Cas9 Protects CD4^+^ T Cells from HIV-1 Infection. Cell Biosci..

[11] Dar A., Kollet O., Lapidot T. (2006). Mutual, Reciprocal SDF-1/CXCR4 Interactions between Hematopoietic and Bone Marrow Stromal Cells Regulate Human Stem Cell Migration and Development in NOD/SCID Chimeric Mice. Exp. Hematol..

[12] Nie Y., Han Y.-C., Zou Y.-R. (2008). CXCR4 Is Required for the Quiescence of Primitive Hematopoietic Cells. J. Exp. Med..

[13] Pham Q.T., Bouchard A., Grütter M.G., Berthoux L. (2010). Generation of Human TRIM5α Mutants with High HIV-1 Restriction Activity. Gene Ther..

[14] Anderson J., Akkina R. (2008). Human Immunodeficiency Virus Type 1 Restriction by Human–Rhesus Chimeric Tripartite Motif 5α (TRIM 5α) in CD34^+^ Cell-Derived Macrophages In Vitro and in T Cells In Vivo in Severe Combined Immunodeficient (SCID-Hu) Mice Transplanted with Human Fetal Tissue. Hum. Gene Ther..

[15] Jung U., Urak K., Veillette M., Nepveu-Traversy M.-É., Pham Q.T., Hamel S., Rossi J.J., Berthoux L. (2015). Preclinical Assessment of Mutant Human TRIM5α as an Anti-HIV-1 Transgene. Hum. Gene Ther..

[16] Delville M., Touzot F., Couzin C., Hmitou I., Djerroudi L., Ouedrani A., Lefrère F., Tuchman-Durand C., Mollet C., Fabreguettes J.-R. (2019). Safety of CD34^+^ Hematopoietic Stem Cells and CD4^+^ T Lymphocytes Transduced with LVsh5/C46 in HIV-1 Infected Patients with High-Risk Lymphoma. Mol. Ther. Methods Clin. Dev..

[17] Burke B.P., Levin B.R., Zhang J., Sahakyan A., Boyer J., Carroll M.V., Colón J.C., Keech N., Rezek V., Bristol G. (2015). Engineering Cellular Resistance to HIV-1 Infection In Vivo Using a Dual Therapeutic Lentiviral Vector. Mol. Ther. Nucleic Acids.

[18] Peterson C.W., Haworth K.G., Burke B.P., Polacino P., Norman K.K., Adair J.E., Hu S.-L., Bartlett J.S., Symonds G.P., Kiem H.-P. (2016). Multilineage Polyclonal Engraftment of Cal-1 Gene-Modified Cells and in Vivo Selection after SHIV Infection in a Nonhuman Primate Model of AIDS. Mol. Ther. Methods Clin. Dev..

[19] Maslennikova A., Kruglova N., Kalinichenko S., Komkov D., Shepelev M., Golubev D., Siniavin A., Vzorov A., Filatov A., Mazurov D. (2022). Engineering T-Cell Resistance to HIV-1 Infection via Knock-In of Peptides from the Heptad Repeat 2 Domain of Gp41. mBio.

[20] Pu J., Wang Q., Xu W., Lu L., Jiang S. (2019). Development of Protein- and Peptide-Based HIV Entry Inhibitors Targeting Gp120 or Gp41. Viruses.

[21] Xiong S., Borrego P., Ding X., Zhu Y., Martins A., Chong H., Taveira N., He Y. (2017). A Helical Short-Peptide Fusion Inhibitor with Highly Potent Activity against Human Immunodeficiency Virus Type 1 (HIV-1), HIV-2, and Simian Immunodeficiency Virus. J. Virol..

[22] Tang X., Jin H., Chen Y., Li L., Zhu Y., Chong H., He Y. (2019). A Membrane-Anchored Short-Peptide Fusion Inhibitor Fully Protects Target Cells from Infections of Human Immunodeficiency Virus Type 1 (HIV-1), HIV-2, and Simian Immunodeficiency Virus. J. Virol..

[23] Popov P., Kalinin R., Buslaev P., Kozlovskii I., Zaretckii M., Karlov D., Gabibov A., Stepanov A. (2023). Unraveling Viral Drug Targets: A Deep Learning-Based Approach for the Identification of Potential Binding Sites. Brief. Bioinform..

[24] Tarasevich A., Filatov A., Pichugin A., Mazurov D. (2015). Monoclonal Antibody Profiling of Cell Surface Proteins Associated with the Viral Biofilms on HTLV-1 Transformed Cells. Acta Virol..

[25] Mazurov D., Ilinskaya A., Heidecker G., Lloyd P., Derse D. (2010). Quantitative Comparison of HTLV-1 and HIV-1 Cell-to-Cell Infection with New Replication Dependent Vectors. PLoS Pathog..

[26] Kalinichenko S., Komkov D., Mazurov D. (2022). HIV-1 and HTLV-1 Transmission Modes: Mechanisms and Importance for Virus Spread. Viruses.

[27] Shunaeva A., Potashnikova D., Pichugin A., Mishina A., Filatov A., Nikolaitchik O., Hu W.-S., Mazurov D. (2015). Improvement of HIV-1 and Human T Cell Lymphotropic Virus Type 1 Replication-Dependent Vectors via Optimization of Reporter Gene Reconstitution and Modification with Intronic Short Hairpin RNA. J. Virol..

[28] Sato K., Aoki J., Misawa N., Daikoku E., Sano K., Tanaka Y., Koyanagi Y. (2008). Modulation of Human Immunodeficiency Virus Type 1 Infectivity through Incorporation of Tetraspanin Proteins. J. Virol..

[29] Milone M.C., O’Doherty U. (2018). Clinical Use of Lentiviral Vectors. Leukemia.

[30] Qin J.Y., Zhang L., Clift K.L., Hulur I., Xiang A.P., Ren B.-Z., Lahn B.T. (2010). Systematic Comparison of Constitutive Promoters and the Doxycycline-Inducible Promoter. PLoS ONE.

[31] Kim J.H., Lee S.-R., Li L.-H., Park H.-J., Park J.-H., Lee K.Y., Kim M.-K., Shin B.A., Choi S.-Y. (2011). High Cleavage Efficiency of a 2A Peptide Derived from Porcine Teschovirus-1 in Human Cell Lines, Zebrafish and Mice. PLoS ONE.

[32] Livak K.J., Schmittgen T.D. (2001). Analysis of Relative Gene Expression Data Using Real-Time Quantitative PCR and the 2^−ΔΔCT^ Method. Methods.

[33] Radtke S., Adair J.E., Giese M.A., Chan Y.-Y., Norgaard Z.K., Enstrom M., Haworth K.G., Schefter L.E., Kiem H.-P. (2017). A Distinct Hematopoietic Stem Cell Population for Rapid Multilineage Engraftment in Nonhuman Primates. Sci. Transl. Med..

[34] Radtke S., Pande D., Cui M., Perez A.M., Chan Y.-Y., Enstrom M., Schmuck S., Berger A., Eunson T., Adair J.E. (2020). Purification of Human CD34^+^CD90^+^ HSCs Reduces Target Cell Population and Improves Lentiviral Transduction for Gene Therapy. Mol. Ther. Methods Clin. Dev..

[35] Liu Y., Geng N., Huang X. (2024). Molecular Regulators of Chemotaxis in Human Hematopoietic Stem Cells. Biochem. Soc. Trans..

[36] Omer-Javed A., Pedrazzani G., Albano L., Ghaus S., Latroche C., Manzi M., Ferrari S., Fiumara M., Jacob A., Vavassori V. (2022). Mobilization-Based Chemotherapy-Free Engraftment of Gene-Edited Human Hematopoietic Stem Cells. Cell.

[37] Miyazawa H., Wada T. (2021). Reversion Mosaicism in Primary Immunodeficiency Diseases. Front. Immunol..

[38] Leslie G.J., Wang J., Richardson M.W., Haggarty B.S., Hua K.L., Duong J., Secreto A.J., Jordon A.P.O., Romano J., Kumar K.E. (2016). Potent and Broad Inhibition of HIV-1 by a Peptide from the Gp41 Heptad Repeat-2 Domain Conjugated to the CXCR4 Amino Terminus. PLoS Pathog..

[39] Louache F., Debili N., Marandin A., Coulombel L., Vainchenker W. (1994). Expression of CD4 by Human Hematopoietic Progenitors [See Comments]. Blood.

[40] Hinckley-Boned A., Barbero-Jiménez C., Tristán-Manzano M., Maldonado-Perez N., Hudecek M., Justicia-Lirio P., Martin F. (2025). Tailoring CAR Surface Density and Dynamics to Improve CAR-T Cell Therapy. J. Immunother. Cancer.

[41] Majumdar S., Murphy P.M. (2018). Adaptive Immunodeficiency in WHIM Syndrome. Int. J. Mol. Sci..

[42] Mazalo J.K., Tay S.S., Kempe D., Biro M. (2024). Chemokine Receptor Distribution on the Surface of Repolarizing T Cells. Biophys. J..

[43] McDermott D.H., Gao J.-L., Liu Q., Siwicki M., Martens C., Jacobs P., Velez D., Yim E., Bryke C.R., Hsu N. (2015). Chromothriptic Cure of WHIM Syndrome. Cell.

[44] Luz M., Fiandaca M.S., Bankiewicz K.S. (2026). Clinical GDNF Delivery Methods for Parkinson’s Disease. J. Park. Dis..

[45] Palasz E., Wysocka A., Gasiorowska A., Chalimoniuk M., Niewiadomski W., Niewiadomska G. (2020). BDNF as a Promising Therapeutic Agent in Parkinson’s Disease. Int. J. Mol. Sci..

