# Supplementary material for: Bicistronic Lentiviral Architecture Increases Surface Density of Membrane-Associated HIV Entry Inhibitors and Confers Tropism-Independent Protection to Modified Cells
Source: Cells. 2026 Sep 19;15(18):1704. doi: 10.3390/cells15181704 (PMC13605048; doi:10.3390/cells15181704)
Supplement: Supplementary file 1 [file cells-15-01704-s001.zip › cells-4540839-supplementary.pdf]

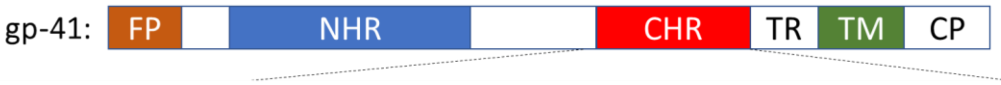


```
        …HTTWMEWDREINNYTSLIHSLIEESQNQQEKNEQELLELDKWASLWNWF…
T20:                   YTSLIHSLIEESQNQQEKNEQELLELDKWASLWNWF
MT-C34:   MTWMEWDREINNYTSLIHSLIEESQNQQEKNEQELL
2P23:    EMTWEEWEKKVEELEKKIEELLK
```

Supplementary Figure S1. Alignment scheme of C-peptides T20, 2P23, and MT-C34 to the HIV-1 gp41 sequence. Abbreviations: FP, fusion peptide; CHR, conserved heptad repeat; NHR, N-terminal heptad repeat; TM, transmembrane domain; CP, cytoplasmic domain.

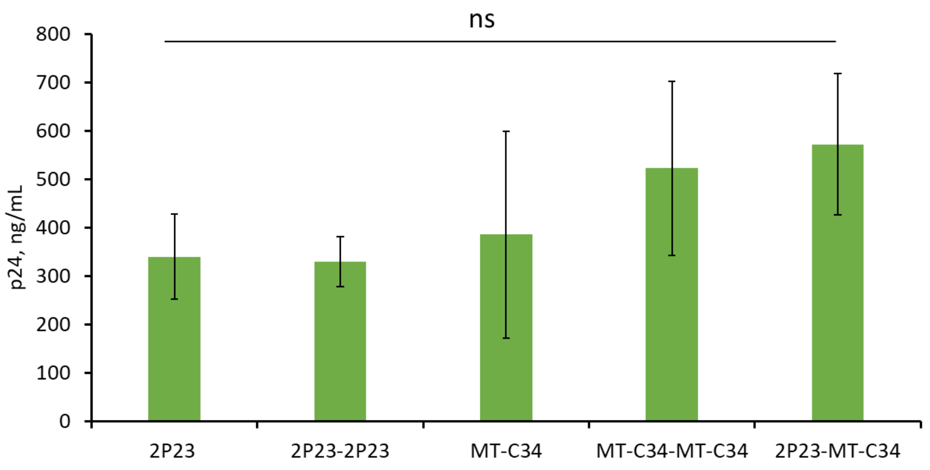

Supplementary Figure S2. Viral titers produced with lentivectors carrying different gene therapy constructs. Mean values ± standard deviation are presented. Mean values were compared by two-tailed Student's t-test, ns – not statistically significant.

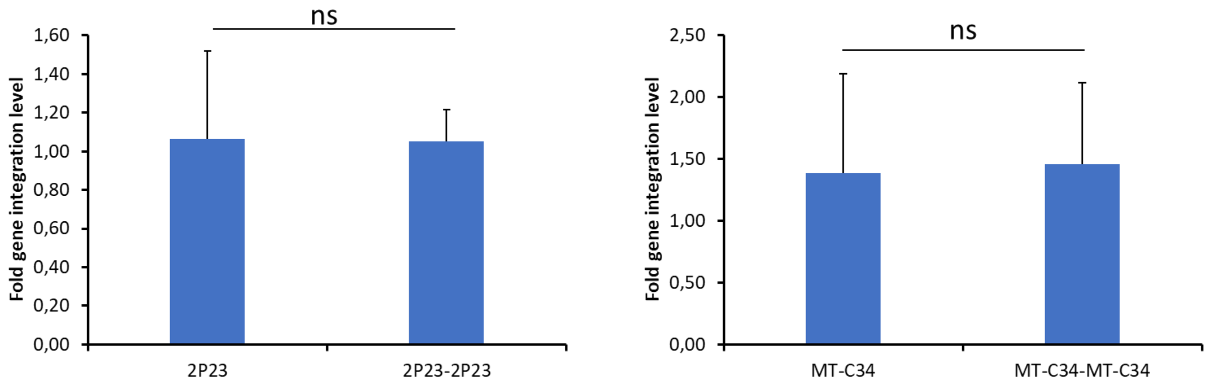

Supplementary Figure S3. The integration level was estimated by Delta Delta (ΔΔCt) Method for Relative Quantification and was equal in cell lines with 2P23 and 2P23-2P23 constructs and with MT-C34 and MT-C34-MT-C34 constructs. Mean values ± standard deviation for three independent measurements are presented. Mean values were compared by two-tailed Student's t-test, ns – not statistically significant.

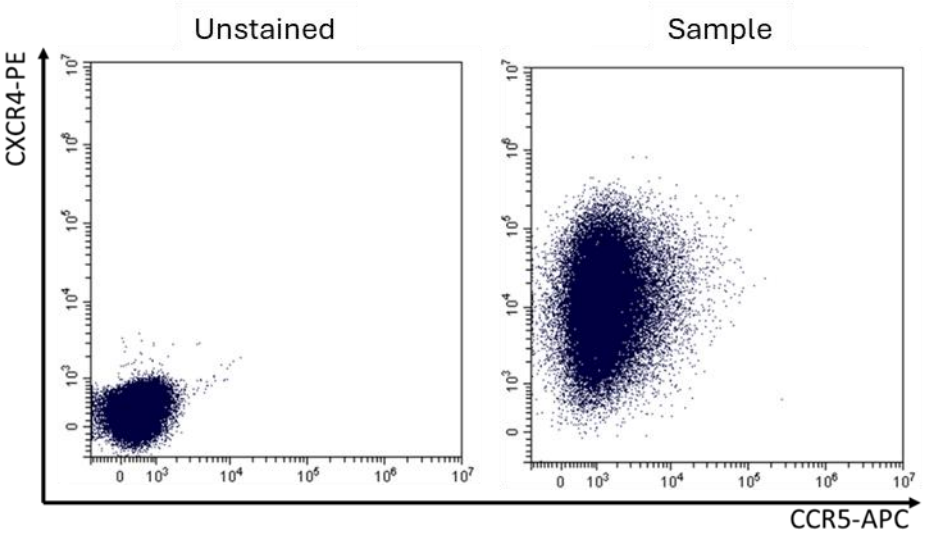

Supplementary Figure S4. Quantification of CXCR4 and CCR5 surface expression levels in CEM/R5 cells via flow cytometry. Histograms represent unstained control and antibody-stained sample.

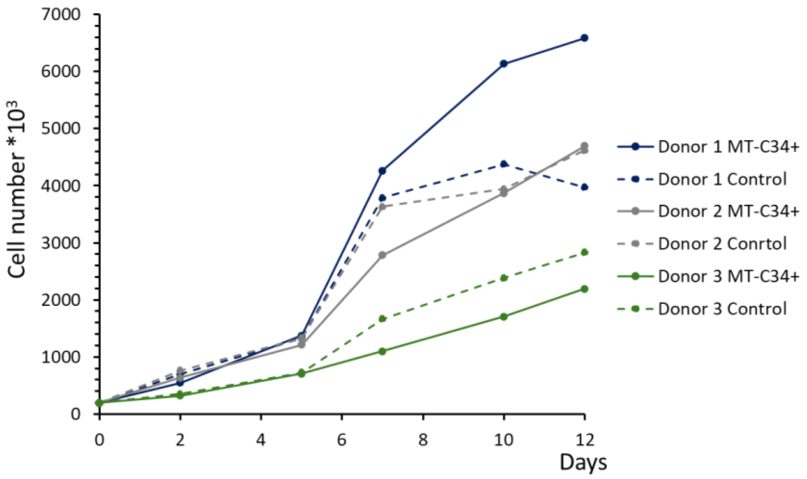

Supplementary Figure S5. Dynamics of CD4+ lymphocyte growth. Day 0 - lentiviral transduction. Mean values for three donors are presented.

A

	I	II	III
SCF	100	100	50
Flt-3 ligand	100	100	50
TPO	10	20	20
IL-6	60	20	50

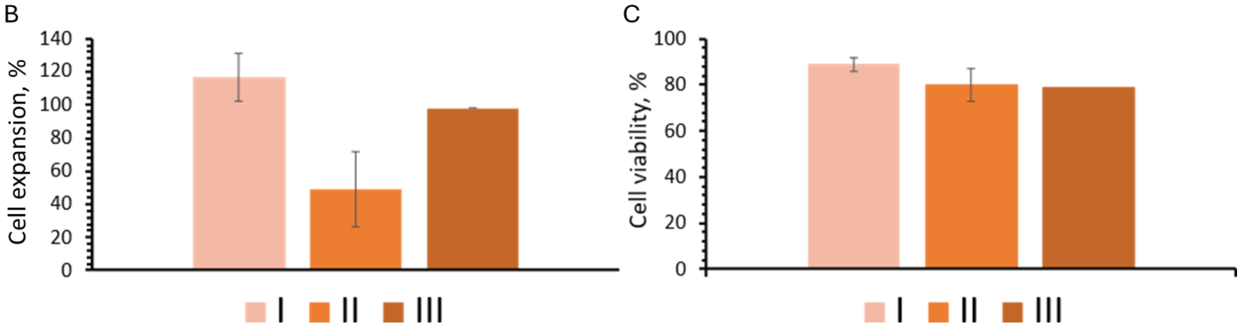

Supplementary Figure S6. Selection of the optimal ratio of cytokines in the culture medium in an experiment with HSCs from two donors. **(A)** Concentration of cytokines in the culture media, ng/mL (**B**) HSC growth in media supplemented with various cytokine combinations, assessed 24 h after culture initiation; **(C)** Viability of HSC in media with different cytokine compositions 24 h after culture initiation.

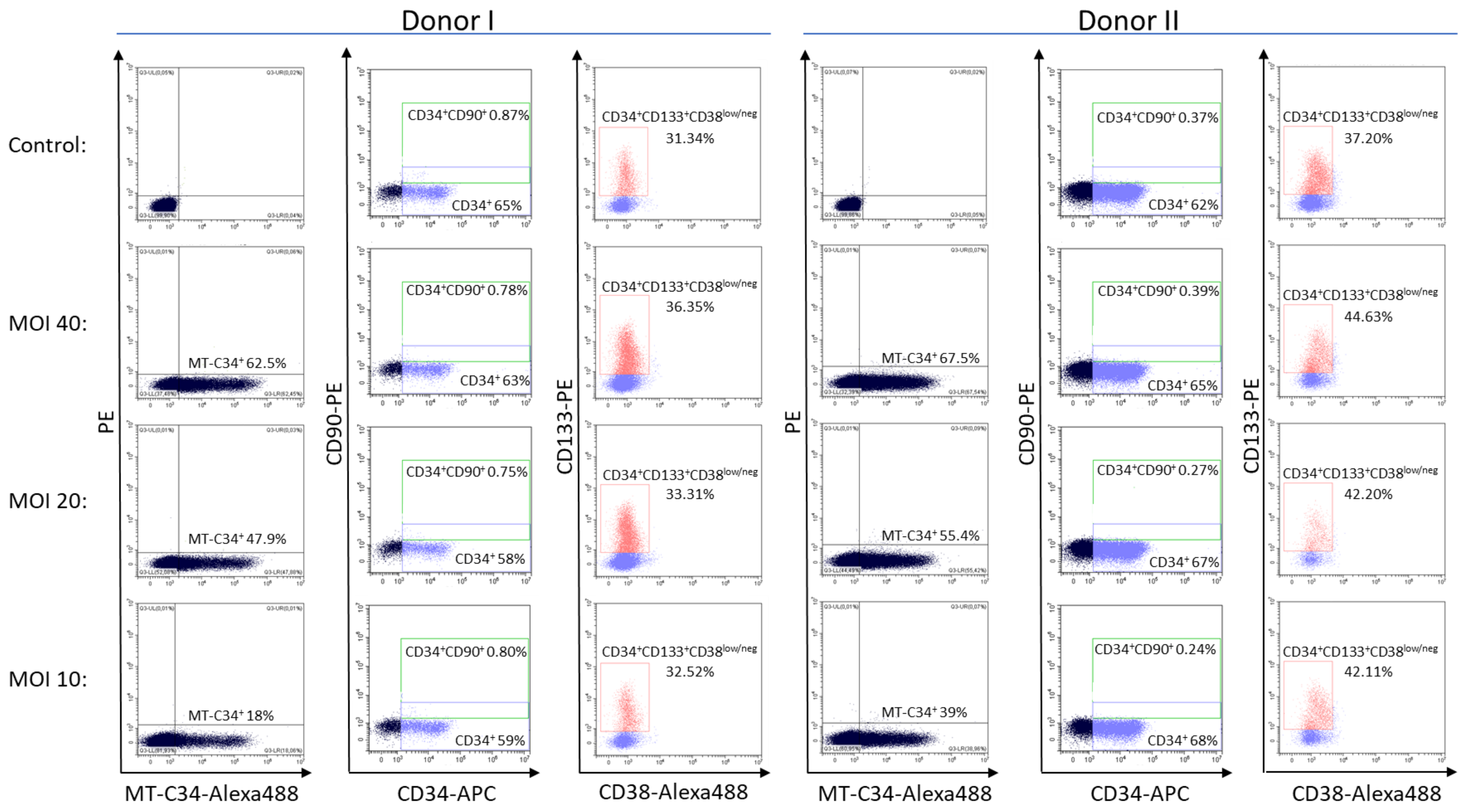
Donor I
Donor II
Control:
MOI 40:
MOI 20:
MOI 10:
PE
CD90-PE
CD133-PE
MT-C34-Alexa488
CD34-APC
CD38-Alexa488
MT-C34+ 62.5%
MT-C34+ 47.9%
MT-C34+ 18%
CD34+CD90+ 0.87%
CD34+ 65%
CD34+CD90+ 0.78%
CD34+ 63%
CD34+CD90+ 0.75%
CD34+ 58%
CD34+CD90+ 0.80%
CD34+ 59%
CD34+CD133+CD38low/neg 31.34%
CD34+CD133+CD38low/neg 36.35%
CD34+CD133+CD38low/neg 33.31%
CD34+CD133+CD38low/neg 32.52%
MT-C34+ 67.5%
MT-C34+ 55.4%
MT-C34+ 39%
CD34+CD90+ 0.37%
CD34+ 62%
CD34+CD90+ 0.39%
CD34+ 65%
CD34+CD90+ 0.27%
CD34+ 67%
CD34+CD90+ 0.24%
CD34+ 68%
CD34+CD133+CD38low/neg 37.20%
CD34+CD133+CD38low/neg 44.63%
CD34+CD133+CD38low/neg 42.20%
CD34+CD133+CD38low/neg 42.11%

Supplementary Figure S7. Analysis of the efficiency of lentiviral transduction and its impact on the expression of hematopoietic stemness markers. The figure shows cytofluorograms of hematopoietic stem cells from two donors transduced with different doses of the MT-C34-MT-C34 lentivirus. The left cytofluorograms show staining data with antibodies against MT-C34, indicating the percentage of MT-C34-positive events. The middle and right cytofluorograms show staining data with antibodies against human CD34, CD133, CD90 and CD38: the middle ones show $CD34^+$ and $CD34^+CD90^+$ events, and the right ones show staining data for CD133 and CD38 from the corresponding gate of $CD34^+$ events. Immunofluorescence staining was performed on day 7 after lentiviral transduction. Each gate analyzed in the cytofluorograms contained at least 1,500 events.

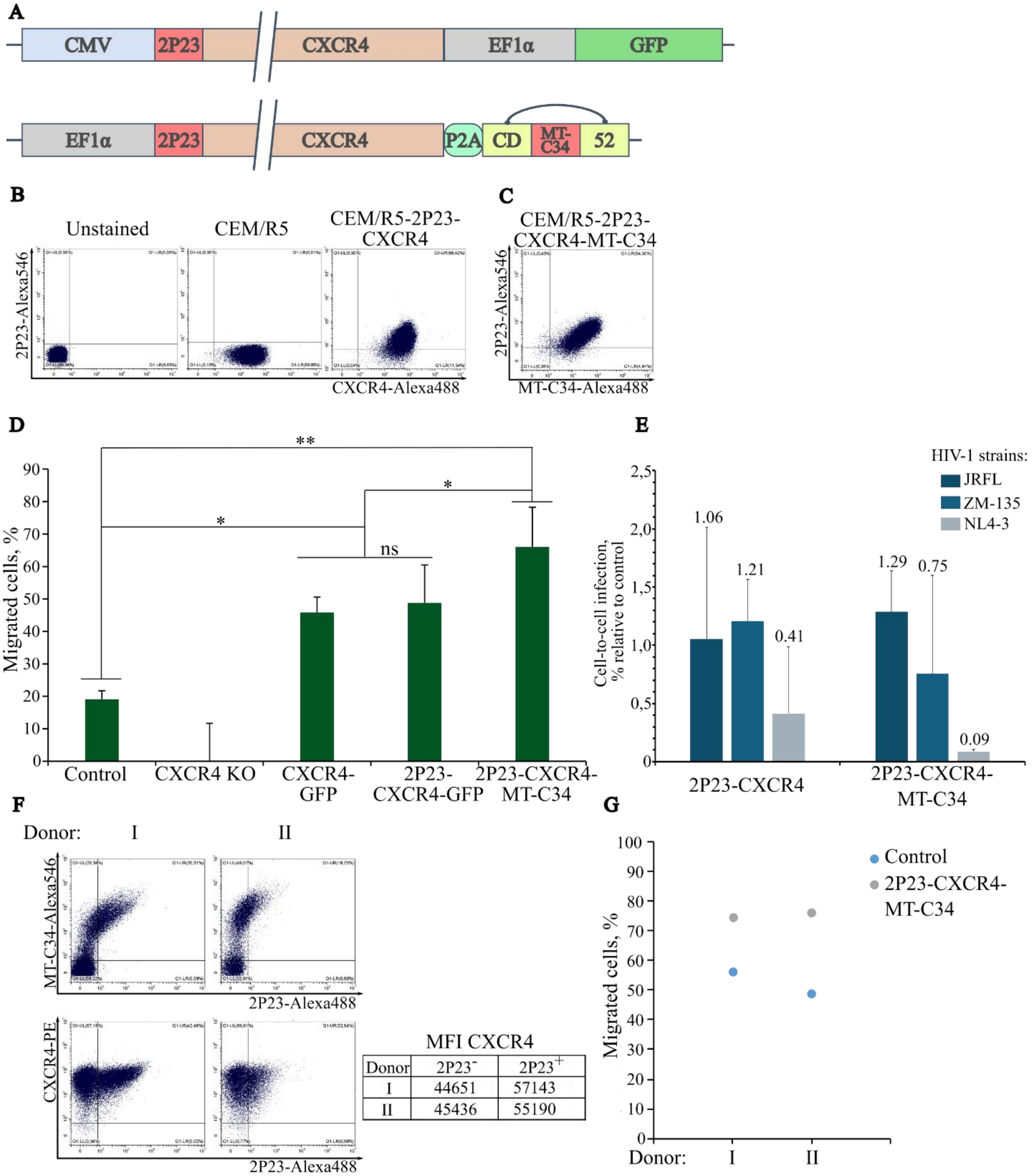

Supplementary Figure S8. Functional characterization of lentivirally delivered CXCR4–2P23 fusion constructs. **(A)** Schematic representation of therapeutic cassettes introduced via lentiviral transduction; **(B, C)** Flow cytometric analysis of CEM/R5 cells transduced with the 2P23–CXCR4–MT-C34 construct and enriched to high purity by fluorescence-activated cell sorting. Panels demonstrate co-expression of 2P23 with surface CXCR4; **(B)** and 2P23 with membrane-localized MT-C34 **(C)**; **(D)** Chemotactic migration of engineered cell lines toward SDF-1. N-terminal fusion of 2P23 does not impair CXCR4-mediated chemotaxis; migration is significantly enhanced when driven by the EF1α promoter relative to the CMV promoter; **(E)** Antiviral protection in cell-to-cell HIV-1 transmission assays using viruses of distinct tropisms. Transduced cells exhibit robust resistance independent of viral tropism. Untransduced CEM/R5 cells were normalized to 100% infection; **(F)** Flow cytometric profiling of primary CD4^{+} T lymphocytes from two healthy donors following transduction with 2P23–CXCR4–MT-C34. Top panels show co-expression of

2P23 and MT-C34; bottom panels show co-expression of 2P23 and CXCR4. Tabulated data indicate elevated CXCR4 MFI in 2P23^{+} and 2P23^{-} subsets, confirming successful construct integration and expression; **(G)** Chemotactic activity of genetically modified CD4^{+} T cells. Cells were sorted based on MT-C34 expression prior to assay. Data represent the percentage of migrated cells relative to input. Modified lymphocytes exhibit enhanced SDF-1-directed migration compared to autologous unmodified controls across both donors.

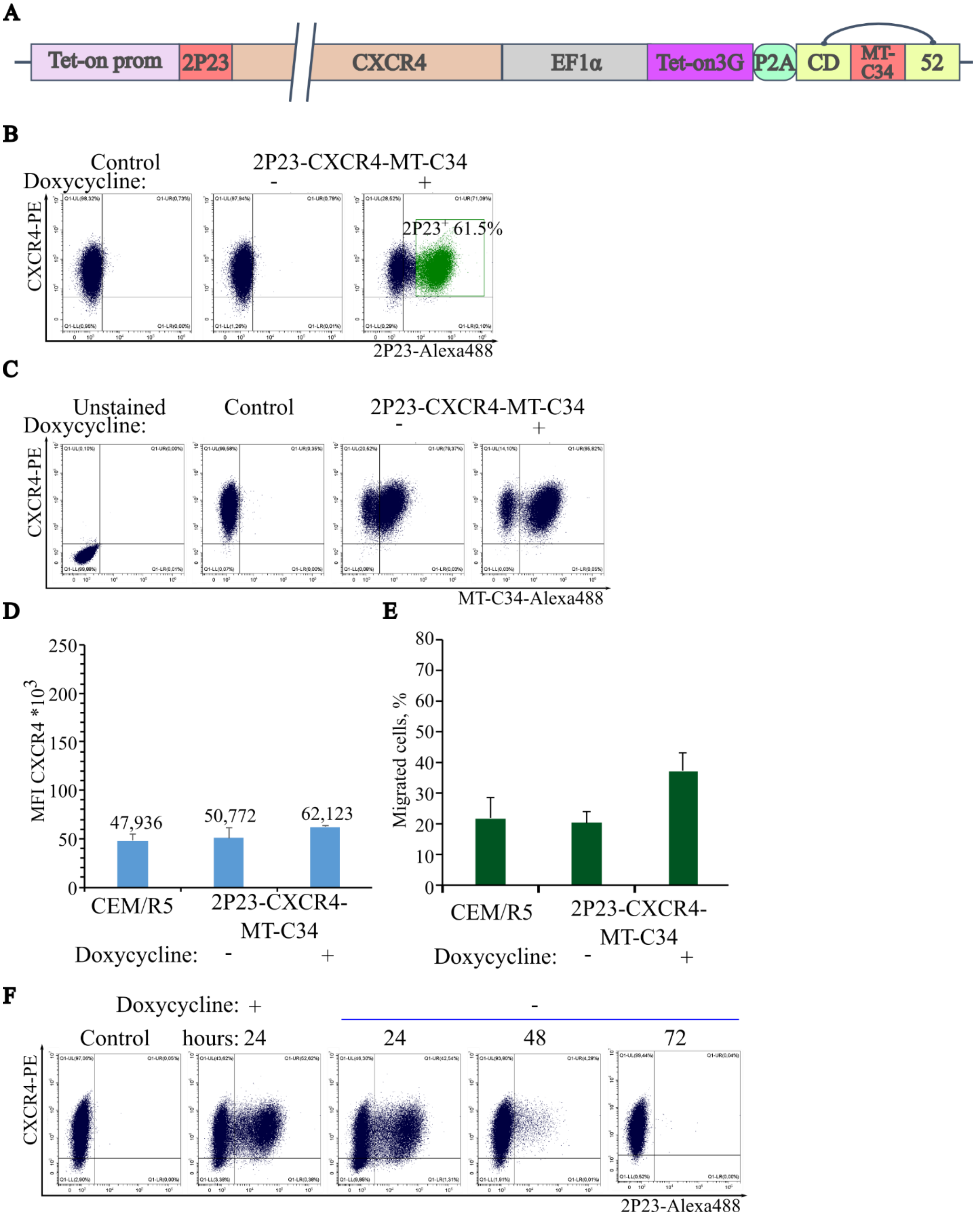

Supplementary Figure S9. Functional characterization of the doxycycline-inducible 2P23–CXCR4 lentiviral construct. **(A)** Schematic representation of the genetic cassette delivered via lentiviral transduction; **(B)** Flow cytometric profiling of 2P23 and CXCR4 expression following doxycycline induction. Transduced cells were purified by sorting for constitutively expressed surface MT-C34 prior to analysis. Doxycycline treatment induces robust 2P23 expression and yields a measurable increase in surface CXCR4; **(C)** Assessment of read-through transcriptional activation of the downstream MT-C34 cassette. Doxycycline induction elevates surface MT-C34 levels beyond baseline constitutive expression, confirming successful read-through promoter architecture; **(D)** Quantitative comparison of CXCR4 MFI in control

CEM/R5 cells versus the engineered line, with and without doxycycline. Data represent three independent experiments ± SD. Although statistical significance was not reached, doxycycline-treated transduced cells exhibit a consistent upward trend in CXCR4 MFI relative to uninduced controls; **(E)** Chemotactic response of control and engineered cells toward SDF-1. Doxycycline-induced cells demonstrate an enhanced migratory tendency compared to uninduced counterparts; however, the difference does not reach statistical significance (p = 0.052); **(F)** Kinetics of transgene downregulation following doxycycline removal. 2P23 expression remains stable at 24 h post-withdrawal, declines markedly by 48 h, and returns to baseline levels by 72 h, demonstrating reversible regulation of the inducible system.

Supplementary Table S1. Description of the gene therapy sequences.

Constructions	Therapeutic sequence[1]
pCDH-MT-C34	atgaagcgcttcctcttcctcctactcaccatcagcctcctggttatggtacagatacaaactggactctcaATGACCTGGATGGAGTGGGACCGGGAGATCAATAACTACACCTCACTGATCCACAGCCTGATCGAGGAGTCCCAGAACCAGCAGGAGAAGAACGAGCAGGAGCTCCTGtcagcatccagcaacataagcggaggcattttcctttcttcgtggccaatgccataatccacctcttctgcttcagttga
pCDH-MT-C34-MT-C34	atgaagcgcttcctcttcctcctactcaccatcagcctcctggttatggtacagatacaaactggactctcaATGACCTGGATGGAGTGGGACCGGGAGATCAATAACTACACCTCACTGATCCACAGCCTGATCGAGGAGTCCCAGAACCAGCAGGAGAAGAACGAGCAGGAGCTCCTGtcagcatccagcaacataagcggaggcattttcctttcttcgtggccaatgccataatccacctcttctgcttcagt**GGATCCGGCGCAACAAACTTCTCTCTGCTGAAACAAGCCGGAGATGTCGAAGAGAATCCTGGACCG**aagcgcttcctcttcctcctactcaccatcagcctcctggttatggtacagatacaaactggactctcaATGACCTGGATGGAGTGGGACCGGGAGATCAATAACTACACCTCACTGATCCACAGCCTGATCGAGGAGTCCCAGAACCAGCAGGAGAAGAACGAGCAGGAGCTCCTGtcagcatccagcaacataagcggaggcattttcctttcttcgtggccaatgccataatccacctcttctgcttcagttga
pCDH-2P23	atgaagcgcttcctcttcctcctactcaccatcagcctcctggttatggtacagatacaaactggactctcaGAGATGACCTGGGAAGAGTGGGAAAAGAAGGTCGAGGAACTGGAGAAGAAAATCGAAGAGCTGCTCAAGtcagcatccagcaacataagcggaggcattttcctttcttcgtggccaatgccataatccacctcttctgcttcagttga
pCDH-2P23-2P23	atgaagcgcttcctcttcctcctactcaccatcagcctcctggttatggtacagatacaaactggactctcaGAGATGACCTGGGAAGAGTGGGAAAAGAAGGTCGAGGAACTGGAGAAGAAAATCGAAGAGCTGCTCAAGtcagcatccagcaacataagcggaggcattttcctttcttcgtggccaatgccataatccacctcttctgcttcagt**GGATCCGGCGCAACAAACTTCTCTCTGCTGAAACAAGCCGGAGATGTCGAAGAGAATCCTGGACCG**aagcgcttcctcttcctcctactcaccatcagcctcctggttatggtacagatacaaactggactctcaGAGATGACCTGGGAAGAGTGGGAAAAGAAGGTCGAGGAACTGGAGAAGAAAATCGAAGAGCTGCTCAAGtcagcatccagcaacataagcggaggcattttcctttcttcgtggccaatgccataatccacctcttctgcttcagctag
pCDH-2P23-MT-C34	atgaagcgcttcctcttcctcctactcaccatcagcctcctggttatggtacagatacaaactggactctcaGAGATGACCTGGGAAGAGTGGGAAAAGAAGGTCGAGGAACTGGAGAAGAAAATCGAAGAGCTGCTCAAGtcagcatccagcaacataagcggaggcattttcctttcttcgtggccaatgccataatccacctcttctgcttcagt**GGATCCGGCGCAACAAACTTCTCTCTGCTGAAACAAGCCGGAGATGTCGAAGAGAATCCTGGACCG**aagcgcttcctcttcctcctactcaccatcagcctcctggttatggtacagatacaaactggactctcaATGACCTGGATGGAGTGGGACCGGGAGATCAATAACTACACCTCACTGATCCACAGCCTGATCGAGGAGTCCCAGAACCAGCAGGAGAAGAACGAGCAGGAGCTCCTGtcagcatccagcaacataagcggaggcattttcctttcttcgtggccaatgccataatccacctcttctgcttcagttga

pCDH-2P23-CXCR4	ATGGAGATGACCTGGGAAGAGTGGGAAAAGAAGGTCGAGGAACTG<br>GAGAAGAAAATCGAAGAGCTGCTCAAGGAGGGGATCAGTATATACA<br>CTTCAGATAACTACACCGAGGAAATGGGCTCAGGGGACTATGACTCC<br>ATGAAGGAACCCTGTTTCCGTGAAGAAAATGCTAATTTCAATAAAAT<br>CTTCCTGCCCACCATCTACTCCATCATCTTCTTAACTGGCATTGTGGG<br>CAATGGATTGGTCATCCTGGTCATGGGTTACCAGAAGAAACTGAGAA<br>GCATGACGGACAAGTACAGGCTGCACCTGTCAGTGGCCGACCTCCT<br>CTTTGTCATCACGCTTCCCTTCTGGGCAGTTGATGCCGTGGCAAACTG<br>GTACTTTGGGAACTTCCTATGCAAGGCAGTCCATGTCATCTACACAGT<br>CAACCTCTACAGCAGTGTCCTCATCCTGGCCTTCATCAGTCTGGACC<br>GCTACCTGGCCATCGTCCACGCCACCAACAGTCAGAGGCCAAGGAA<br>GCTGTTGGCTGAAAAGGTGGTCTATGTTGGCGTCTGGATCCCTGCCCT<br>CCTGCTGACTATTCCCGACTTCATCTTTGCCAACGTCAGTGAGGCAG<br>ATGACAGATATATCTGTGACCGCTTCTACCCCAATGACTTGTGGGTGG<br>TTGTGTTCCAGTTTCAGCACATCATGGTTGGCCTTATCCTGCCTGGTAT<br>TGTCATCCTGTCCTGCTATTGCATTATCATCTCCAAGCTGTCACACTCC<br>AAGGGCCACCAGAAGCGCAAGGCCCTCAAGACCACAGTCATCCTC<br>ATCCTGGCTTTCTTCGCCTGTTGGCTGCCTTACTACATTGGGATCAGC<br>ATCGACTCCTTCATCCTCCTGGAAATCATCAAGCAAGGGTGTGAGTT<br>TGAGAACACTGTGCACAAGTGGATTTCCATCACCGAGGCCCTAGCTT<br>TCTTCCACTGTTGTCTGAACCCCATCCTCTATGCTTTCCTTGGAGCCA<br>AATTTAAAACCTCTGCCCAGCACGCACTCACCTCTGTGAGCAGAGG<br>GTCCAGCCTCAAGATCCTCTCCAAAGGAAAGCGAGGTGGACATTCA<br>TCTGTTTCCACTGAGTCTGAGTCTTCAAGTTTTCACTCCAGCTAACAC<br>AGATGTAAAAGACTTTTTTTATACGATAAATAACTTTTTTTTAAGTTA<br>CACATTTTTCAGATATAAAAGACTGACCAATATTGTACAGTTTTTATTG<br>CTTGTTGGATTTTTGTCTTGTGTTTCTTTAGTTTTTGTGAAGTTTAATTG<br>ACTTATTTATATAAATTTTTTTTGTTTCATATTGATGTGTGTCTAGGCAG<br>GACCTGTGGCCAAGTTCTTAGTTGCTGTATGTCTCGTGGTAGGACTGT<br>AGAAAAGGGAACTGAACATTCCAGAGCGTGTAGTGAATCACGTAAA<br>GCTAGAAATGATCCCCAGCTGTTTATGCATAGATAATCTCTCCATTCC<br>CGTGGAACGTTTTTCCTGTTCTTAAGACGTGATTTTGCTGTAGAAGAT<br>GGCACTTATAACCAAAGCCCAAAGTGGTATAGAAATGCTGGTTTTTC<br>AGTTTTCAGGAGTGGGTTGATTTCAGCACCTACAGTGTACAGTCTTGT<br>ATTAAGTTGTTAATAAAAGTACATGTTAAACTTACTTAG
pCDH-2P23-CXCR4-MT-C34	ATGGAGATGACCTGGGAAGAGTGGGAAAAGAAGGTCGAGGAACTG<br>GAGAAGAAAATCGAAGAGCTGCTCAAGGAGGGGATCAGTATATACA<br>CTTCAGATAACTACACCGAGGAAATGGGCTCAGGGGACTATGACTCC<br>ATGAAGGAACCCTGTTTCCGTGAAGAAAATGCTAATTTCAATAAAAT<br>CTTCCTGCCCACCATCTACTCCATCATCTTCTTAACTGGCATTGTGGG<br>CAATGGATTGGTCATCCTGGTCATGGGTTACCAGAAGAAACTGAGAA<br>GCATGACGGACAAGTACAGGCTGCACCTGTCAGTGGCCGACCTCCT<br>CTTTGTCATCACGCTTCCCTTCTGGGCAGTTGATGCCGTGGCAAACTG<br>GTACTTTGGGAACTTCCTATGCAAGGCAGTCCATGTCATCTACACAGT<br>CAACCTCTACAGCAGTGTCCTCATCCTGGCCTTCATCAGTCTGGACC

	GCTACCTGGCCATCGTCCACGCCACCAACAGTCAGAGGCCAAGGAA<br>GCTGTTGGCTGAAAAGGTGGTCTATGTTGGCGTCTGGATCCCTGCCCT<br>CCTGCTGACTATTCCCGACTTCATCTTTGCCAACGTCAGTGAGGCAG<br>ATGACAGATATATCTGTGACCGCTTCTACCCCAATGACTTGTGGGTGG<br>TTGTGTTCCAGTTTCAGCACATCATGGTTGGCCTTATCCTGCCTGGTAT<br>TGTCATCCTGTCCTGCTATTGCATTATCATCTCCAAGCTGTCACACTCC<br>AAGGGCCACCAGAAGCGCAAGGCCCTCAAGACCACAGTCATCCTC<br>ATCCTGGCTTTCTTCGCCTGTTGGCTGCCTTACTACATTGGGATCAGC<br>ATCGACTCCTTCATCCTCCTGGAAATCATCAAGCAAGGGTGTGAGTT<br>TGAGAACACTGTGCACAAGTGGATTTCCATCACCGAGGCCCTAGCTT<br>TCTTCCACTGTTGTCTGAACCCCATCCTCTATGCTTTCCTTGGAGCCA<br>AATTTAAAACCTCTGCCCAGCACGCACTCACCTCTGTGAGCAGAGG<br>GTCCAGCCTCAAGATCCTCTCCAAAGGAAAGCGAGGTGGACATTCA<br>TCTGTTTCCACTGAGTCTGAGTCTTCAAGTTTTCACTCCAGCTAACAC<br>AGATGTAAAAGACTTTTTTTTATACGATAAATAACTTTTTTTTAAGTTA<br>CACATTTTTCAGATATAAAAGACTGACCAATATTGTACAGTTTTTATTG<br>CTTGTTGGATTTTTGTCTTGTGTTTCTTTAGTTTTTGTGAAGTTTAATTG<br>ACTTATTTATATAAATTTTTTTTGTTTCATATTGATGTGTGTCTAGGCAG<br>GACCTGTGGCCAAGTTCTTAGTTGCTGTATGTCTCGTGGTAGGACTGT<br>AGAAAAGGGAACTGAACATTCCAGAGCGTGTAGTGAATCACGTAAA<br>GCTAGAAATGATCCCCAGCTGTTTATGCATAGATAATCTCTCCATTCC<br>CGTGGAACGTTTTTCCTGTTCTTAAGACGTGATTTTGCTGTAGAAGAT<br>GGCACTTATAACCAAAGCCCAAAGTGGTATAGAAATGCTGGTTTTTC<br>AGTTTTCAGGAGTGGGTTGATTTCAGCACCTACAGTGTACAGTCTTGT<br>ATTAAGTTGTTAATAAAAGTACATGTTAAACTTACC**GGTTCCGGCGC**<br>**AACAAACTTCTCTCTGCTGAAACAAGCCGGAGATGTCGAAGAGA**<br>**ATCCTGGACCG**aagcgcttcctcttcctcctactcaccatcagcctcctggttatggtacagatacaaa<br>ctggactctcaATGACCTGGATGGAGTGGGACCGGGAGATCAATAACTAC<br>ACCTCACTGATCCACAGCCTGATCGAGGAGTCCCAGAACCAGCAGG<br>AGAAGAACGAGCAGGAGCTCCTGtcagcatccagcaacataagcggaggcatttcctt<br>tcttcgtggccaatgccataatccacctcttctgcttcagttga

[1]lowercase– export and anchoring signals; UPPERCASE– CXCR4; UNDERLINED UPPERCASE – 2P23 or MT-C34; BOLD UPPERCASE – P2A.

Supplementary Table S2. qPCR primer sequences.

Primer	Subsequence
F-BMP4	CAG GGC TTC CAC CGT ATA AAC
R-BMP4	GTG TCC AGT AGT CGT GTG ATG
F-WPRE	ACG CTG CTT TAA TGC CTT TG
R-WPRE	GGG CCA CAA CTC CTC ATA AA
